# Molecular mechanism of potently neutralizing human monoclonal antibodies against severe fever with thrombocytopenia virus infection

**DOI:** 10.1128/jvi.00533-25

**Published:** 2025-06-20

**Authors:** Chuansong Quan, Kaixiao Nie, Dezhen Ma, Chao Su, Lianfeng Li, Wenjun Zheng, Chunhong Yin, Yiwen Wang, Peipei Yang, Dingkun Peng, Xin Liu, Weiwei Li, Weixiao Liu, Chao Shan, Jie Zheng, Di Liu, Hong Zhang, Michael J. Carr, George F. Gao, Jianxun Qi, Weifeng Shi

**Affiliations:** 1 Key Laboratory of Emerging Infectious Diseases in Universities of Shandong, Shandong First Medical University & Shandong Academy of Medical Sciences, Ji'nan, China; 2 Department of Hematology, The Second Affiliated Hospital of Shandong First Medical University, Taian, China; 3 School of Public Health, Shandong First Medical University & Shandong Academy of Medical Sciences, Ji'nan, China; 4 Department of Vector Control, Taian Center for Disease Control and Prevention, Taian, China; 5 Jiangsu Provincial Key Laboratory of Critical Care Medicine, School of Medicine, Advanced Institute for Life and Health, Southeast University, Nanjing, China; 6 State Key Laboratory for Animal Disease Control and Prevention, Harbin Veterinary Research Institute, Chinese Academy of Agricultural Sciences 111613 , Harbin, China; 7 Infectious Disease Control Institute, Shandong Center for Disease Control and Prevention, Shandong Provincial Key Laboratory of Infectious Disease Prevention and Control, Ji'nan, China; 8 State Key Laboratory of Virology and Biosafety, Wuhan Institute of Virology, Chinese Academy of Sciences 74614 , Wuhan, China; 9 Shanghai Institute of Virology, Shanghai Jiao Tong University School of Medicine 56694, Shanghai, China; 10 CAS Key Laboratory of Pathogen Microbiology and Immunology, Institute of Microbiology, Chinese Academy of Sciences 85387, Beijing, China; 11 Department of Infectious Disease Prevention and Control, Taian Center for Disease Control and Prevention, Taian, China; 12 Key Laboratory of Special Pathogens and Biosafety, Center for Biosafety Mega-Science, Wuhan Institute of Virology, Chinese Academy of Sciences 74614 , Wuhan, China; 13 National Virus Reference Laboratory, School of Medicine, University College Dublin 37438, Dublin, Ireland; 14 International Collaboration Unit, International Institute for Zoonosis Control, Hokkaido University 12810, Sapporo, Japan; 15 Ruijin Hospital, Shanghai Jiao Tong University School of Medicine 56694, Shanghai, China; Lerner Research Institute, Cleveland Clinic, Cleveland, Ohio, USA

**Keywords:** severe fever with thrombocytopenia syndrome, SFTSV, *Bandavirus dabieense*, monoclonal antibodies, neutralization, therapeutics

## Abstract

Although severe fever with thrombocytopenia syndrome (SFTS) was first described in China in 2009, the case fatality rate remains >40% among patients with multi-organ failure. To date, no antivirals specifically targeting SFTSV have been approved. We obtained several monoclonal antibodies (mAbs) from SFTS survivors by single-cell RNA-seq. Neutralization and animal experiments were applied to assess the effects of these mAbs *in vitro* and *in vivo*, and co-crystal structures with SFTSV-Gn glycoproteins were determined by X-ray crystallography. The mAbs SD4, SD12, and SD22 targeted the SFTSV-Gn with high neutralizing activities, and, remarkably, SD4 and SD22 exhibited *K_D_* values in the range of 32–83 pM for different viral genotypes. Notably, a single administration (20 mg/kg) of SD4 and SD22 showed 100% protection in mice at day 3 post-inoculation (dpi). Importantly, SD4 also provided 60% protection at a lower dose (0.3 mg/kg) when administered at 3 dpi. The crystallographic structures of SD4, SD22, and SD12 with Gn were determined at 3.3 Å, 2.8 Å, and 2.4 Å, respectively, which revealed that they recognized a conserved antigenic epitope around the hexon wellhead edge. These human-derived mAbs have significant therapeutic potential for severe SFTS cases and provide a basis for rational antibody-based vaccine designs and clinical trials.

**IMPORTANCE:**

The incidence of severe fever with thrombocytopenia syndrome (SFTS) has been increasing in Asia in recent years; however, no specific antiviral agents have been approved to date. Herein, we report a panel of anti-SFTSV Gn monoclonal antibodies (mAbs) with excellent neutralizing capacities and remarkable therapeutic potential *in vitro* and *in vivo* in a mouse model. In addition, crystallographic structures of mAbs complexed with Gn were resolved with atomic resolution (2.4 Å–3.3 Å), revealing a conserved antigenic epitope near the hexon wellhead edge. In sum, the neutralizing antibodies reported in the present study have significant therapeutic potential, paving the way for effective treatment of severe SFTS patients.

## INTRODUCTION

Severe fever with thrombocytopenia syndrome (SFTS) is an emerging tick-borne zoonotic disease caused by a bunyavirus, SFTS virus (SFTSV). Since SFTSV was first identified in rural areas of central China in 2009 (1), there have been subsequent reports of laboratory-confirmed SFTSV cases in humans in Japan, South Korea, Vietnam, Thailand, and Pakistan and, also, infections in domesticated animals, indicative of a broader threat to global public health (2–6). The overall pooled notification and mortality rates were 18.93 and 3.49 per 10 million individuals, respectively, and the overall pooled case fatality rate (CFR) was 7.80% globally (7). Significantly, the CFR in SFTS patients with multi-organ failure and central nervous system involvement has been reported to be as high as 44.70% (8). This may be attributable to the ability of SFTSV to infect monocytes, differentiated B cells, and platelets (9–12). SFTSV infection of immune cells results in cell lysis and death, releasing both IFN-γ and TNF-α, thereby activating corresponding T lymphocytes, producing large amounts of inflammatory cytokines, and inducing a maladaptive immune response (13).

Several treatments have been applied to SFTS patients (e.g., favipiravir, steroids, intravenous immunoglobulin [IVIG], and therapeutic plasma exchange) (14). Favipiravir has shown potential efficacy in treating SFTS patients when administered early during mild symptomatic stages. However, the eﬃcacy of IVIG and steroids in treating SFTS remains unclear and requires further study. Other small-molecule inhibitors to treat SFTS, such as triclosan, calcium channel blockers, amodiaquine derivatives, and SFTSV-speciﬁc antibodies, are currently under development (14–22). No speciﬁc antiviral drugs have yet proven eﬀective against SFTS disease, and the primary treatment for SFTS is supportive, despite poor clinical efficacy.

The genome of SFTSV is comprised of three L, M, and S gene segments, and the M segment encodes a membrane protein precursor that undergoes proteolytic maturation to produce two glycoproteins: Gn (the N-terminus) and Gc (the C-terminus) embedded in the viral envelope (23). It has been shown previously that Gn plays a major role in binding to host receptors, and a number of effective neutralizing epitopes targeting Gn have been identified (15–22). The monoclonal antibody (mAb) Ab10 and the SNB02 nanobody have been shown to effectively prevent SFTSV infection in murine models; however, the precise antigenic interaction sites are not yet clear (18, 19). The human mAb SF5 binds to subdomain I in the SFTSV Gn head; however, the neutralization potential requires optimization for the prevention of SFTSV infection (15). Recently, the mAbs S2A5 and 40C10 obtained from mice were reported to identify a new neutralizing epitope in the SFTSV-Gn (16, 17). It is important to note, however, that the affinity of the previously reported antibodies to their cognate epitopes is only at the nanomolar scale, and their ability to protect moribund mice four to six days post-infection with a lethal challenge of SFTSV currently remains unclear, which corresponds to the time of clinical presentation of severe and critical SFTSV human cases. Thus, human-derived mAbs with potent neutralization efficacies represent a rational approach to target conserved, druggable epitopes on the surface of the SFTSV Gn and potentially offer a promising new therapeutic option to reduce disease morbidity and mortality in symptomatic patients.

In the present study, we have identified multiple mAbs from B cell immune repertoires in SFTS convalescent patients that possess both potent neutralizing activities and binding affinities. Among them, SD4 and SD22 showed the highest neutralizing activity with half-maximal inhibitory concentration (IC_50_) values ≤ 1 ng/mL, and strong binding affinity with *K_D_*’s in the range of 32–83 pM for different SFTSV genotypes. Strikingly, a single dose of SD4 could afford 100%, 80%, and 20% protection of mice with a lethal dose challenge of SFTSV in groups treated 4, 5, and 6 days post-infection (dpi), respectively. Multiple doses of SD4 could further increase the survival rates of mice in the 5 and 6 dpi groups. Importantly, a reduced dosage (0.3 mg/kg) of SD4 could still afford 60% protection when administered at 3 dpi. We further resolved the antibody-epitope crystallographic structures of SD4, SD22, and SD12 to atomic resolution and found that the amino acids associated with the antibody-epitope interaction were conserved across all known SFTSV genotypes.

## RESULTS

### B-cell immune responses of the recovered SFTS patients

**Fig 1:**
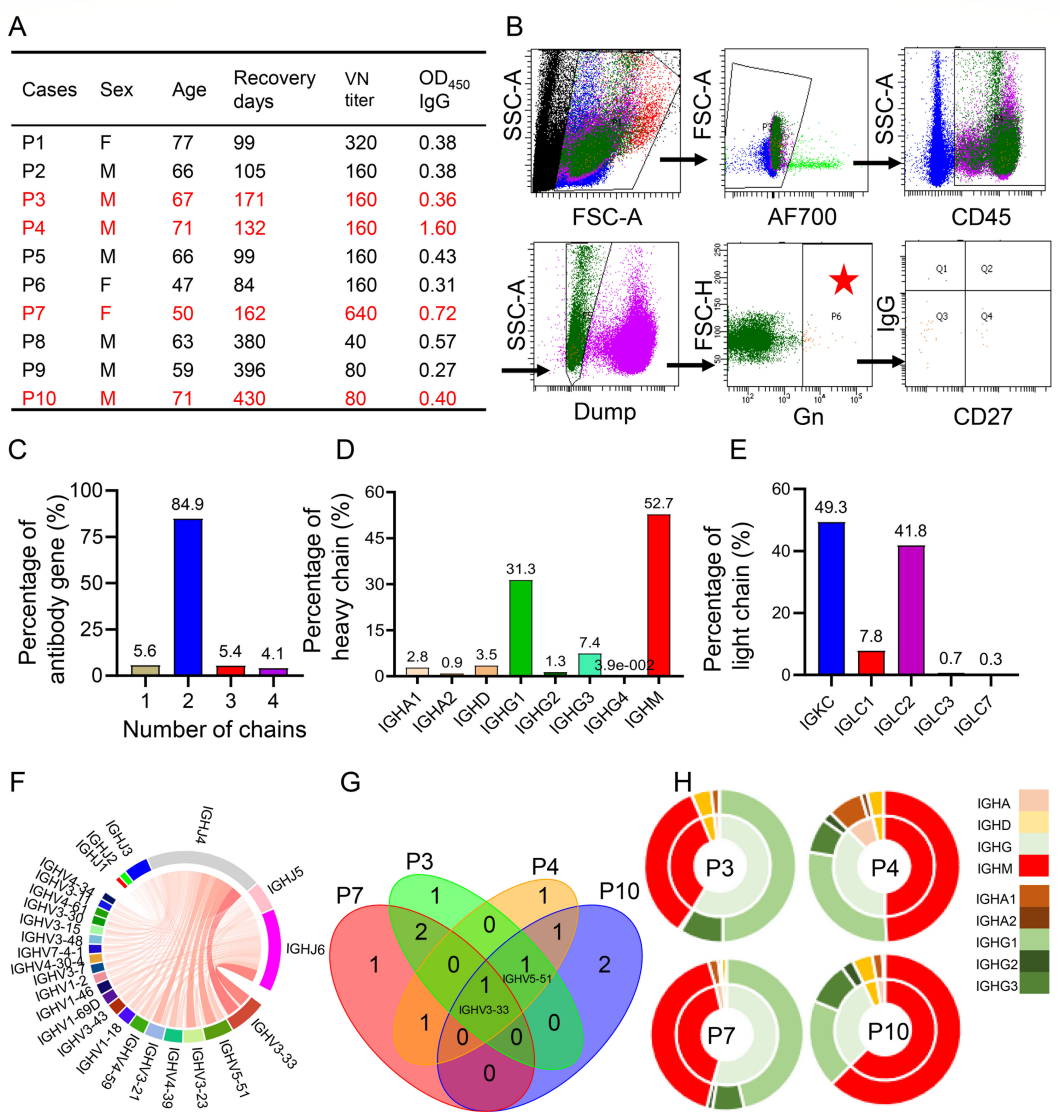
Characterization of SFTSV-Gn specific B cell repertoires from the ten recovered SFTS cases. (**A**) Basic characteristics of the recovered patients. Case sex, age, recovery days (days between SFTS diagnosis and sample collection), the virus neutralization (VN) titer in serum, and OD_450_ measured by ELISA against SFTSV Gn glycoprotein are shown. Subjects highlighted in red font were labeled by hashtag antibodies. (**B**) Representative flow cytometry gating strategy for SFTSV-Gn-specific B cell subpopulations in the blood of the recovered patients. The cells in the red star gate were collected for single-cell RNA sequencing. (**C**) The number of antibody heavy or light chain genes present in each B cell type. (**D and E**) The percentages of different heavy or light chain subtypes in each B cell type. (**F**) Circos plot showing the V-J gene segment rearrangement characteristics in the BCR repertoires from the recovered patients. The top 20 V genes and all of the J genes were shown in the Circos plot. (**G**) Venn diagram depicting the top five overlapping genes among the four BCR repertoires from the recovered patients (P3, P4, P7, and P10). (**H**) Pie charts showing the ratio of IGH types sorted from typical cases, P3, P4, P7, and P10, respectively. The inner circle represents the ratio of IGH types, and the outer circle represents the IGHA and IGHG subtypes. IGHA, IGHD, IGHG, and IGHM are shown in brown, yellow, green, and red, respectively.

To better understand the memory B cell subsets elicited following SFTSV infection in humans, we enrolled ten individuals who had recovered from SFTSV infection between 84 and 430 days. All of them were diagnosed with SFTSV by real-time qRT-PCR. The age ranges were from 47 to 77 years, and 7 (70%) were male (Fig. 1A). We determined IgG titers against the Gn glycoprotein by enzyme-linked immunosorbent assay (ELISA) and serum neutralization in all individuals, and the neutralizing antibody titers were relatively high (≥160) for P1–P7. For the cases P8–P10, who had recovered for longer than 1 year, the neutralizing antibody titers were 40, 80, and 80, respectively, suggesting that the SFTSV neutralizing antibodies could persist for at least 430 days in convalescent SFTS cases (Fig. 1A).

### Profiling of SFTSV-Gn binding B cell subtypes

To profile the SFTSV Gn-specific B cell characteristics at different recovery phases of the 10 SFTS survivors, we isolated and detected memory B cells from peripheral blood mononuclear cells (PBMCs) that bound to the SFTSV-Gn glycoprotein to study their B cell profiles employing single-cell RNA-seq (scRNA-seq) and scV(D)J-seq (Fig. 1B; see also Fig. S1A). Ultimately, all samples obtained from different recovery phases were pooled because of the small number of SFTSV-specific immune cells. Finally, we sorted approximately 50,000 B cells from the 10 individuals that bound to the SFTSV-Gn glycoprotein as determined by fluorescence-activated cell sorting (FACS). We acquired 8,555 single-cell transcriptomes from two pools, including 6,478 B cells with productive V-J spanning pairs after quality filtering, and 84.9% of them had two mRNA chains (Fig. 1C). Of the cells collected from the recovery stage, the highest ratio was found to be of the IgM genotype with an average of up to 52.7% of the total, followed by IGHG1 (31.3%), IGHG3 (7.4%), IGHD (3.5%), IGHA1 (2.8%), IGHG2 (1.3%), IGHA2 (0.9%), and IGHG4 (0.04%; Fig. 1D). IGKC (49.3%) and IGLC2 (41.8%) represented the major light chain types (Fig. 1E). For the V-J pairs, IgGHV3-33, IgGHV5-51, IgGHV3-23, IgGHV4-39, and IgGHV3-21 ranked as the top five (Fig. 1F). However, only IgGHV3-33 and IgGHV5-51 were identified in four and three different individuals, respectively (Fig. 1G). With regard to the antibody heavy chain, the remaining dominant genotypes were considered to be individual-specific in nature. For example, SFTSV-specific IgM memory B cells still predominated in patient P4 (who had recovered for 171 days from initial diagnosis) and, also, patient P10 (recovered for 430 days), whereas patients P3 and P7, who had recovered for 160–170 days, respectively, had switched to IgG clones (Fig. 1H).

To systematically describe the transcriptomic features against SFTSV-specific B and plasma cells, we used uniform manifold approximation and projection (UMAP) for dimensionality reduction and visualization by clustering the cells employing a graph-based method, which yielded seven clusters based on the differentially-expressed genes (Fig. S1B). Specifically, we identified six major known B cell types by their unique signature genes (*CD19*, *CD79A*, *MS4A1*, and *HLA-DRA*), including 2,107 memory B cells (*CD27*, and *TNFRSF13B*), 1,614 naive B cells (*IGHD*, *IGHM*, *TCL1A*, and *BACH2*), 1,103 resting B cells (*RPS27*, *RPL10*, *CD40*, and *HLA-DPB1*), 788 transitional B cells (*CD24*, *CD27*, and *CD38*), 589 switched B cells (*IGHA1*, *IGHG3*, *CXCR3*, and *IL-10RA*), and 177 plasma cells (*XBP1*, *IFR4*, *CD83*, *CXCR5*, and *JCHAIN*; Fig. S1C and D). Antibody sequences from switched B cells, memory cells, or plasma cell types were selected for functional validation.

### Monoclonal antibody screening and identification of SFTSV neutralizing antibodies

A total of 23 mAbs were selected, with 15 antibodies selected based on the unique molecular identifier (UMI) and eight antibodies based on clonotypes (Table S1). The VH and VL sequences of the 23 antibodies were optimized, synthesized, and cloned into a pcDNA3.4 expression plasmid with corresponding constant regions of the H and L chains of human IgG1. Subsequently, all heavy and light chain pairs were transiently expressed in human embryonic kidney (HEK) 293 F cells and screened for binding affinities to SFTSV or the Gn glycoprotein by ELISA assay. Apart from one antibody with low expression levels (SD16 <0.01 mg/L), the remaining 22 antibodies were well expressed and employed for subsequent experiments.

**Fig 2:**
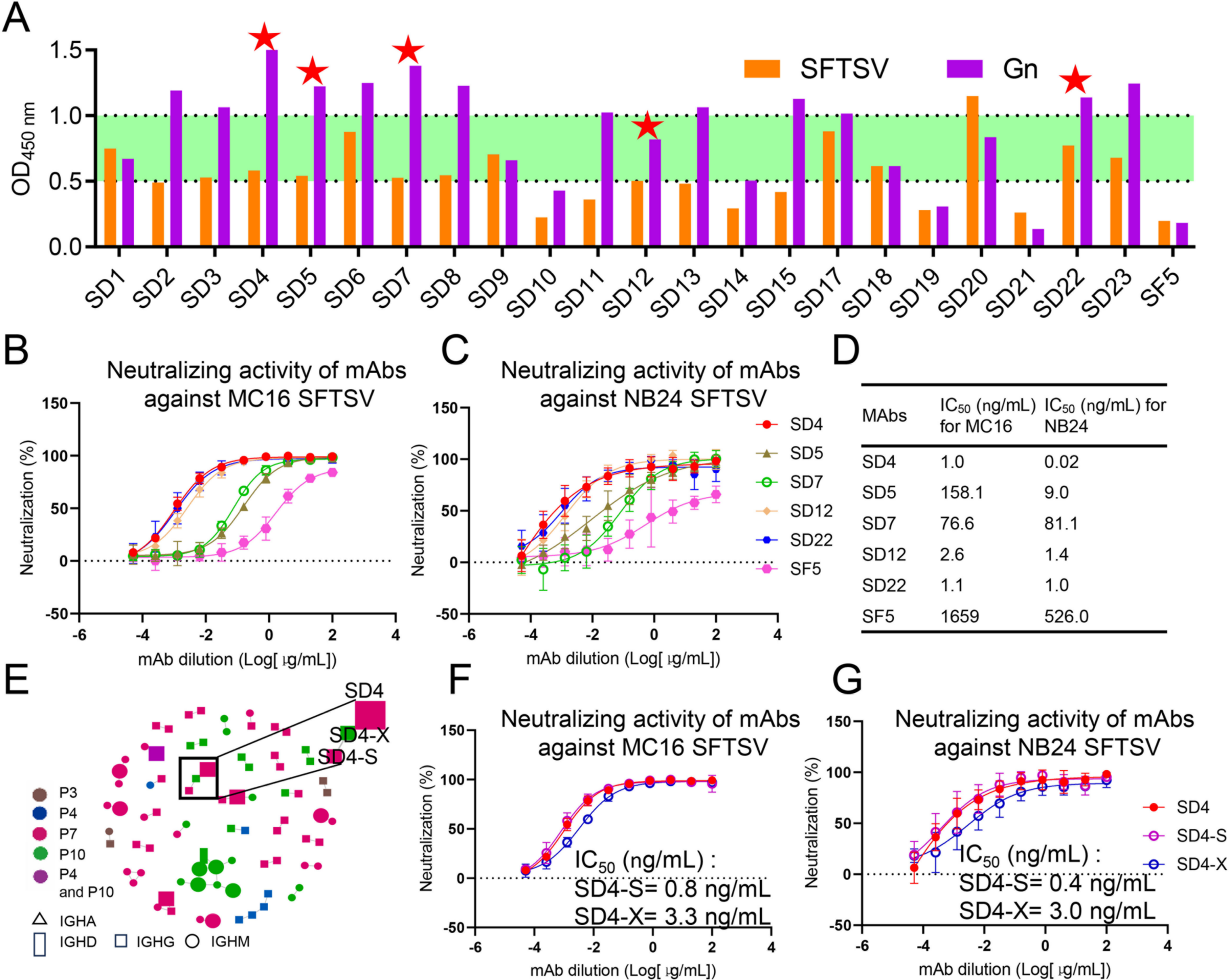
Monoclonal antibody screening and identification of SFTSV neutralizing antibodies. (**A**) ELISA binding of all expressed antibodies (*n* = 22) against Gn and SFTSV. Absorbance at 450 nm was recorded. Experiments were performed twice, and average values are shown. Five mAbs determined to be positive in the neutralization experiments are highlighted with red stars. The SF5 antibody was employed as a positive control. (**B–D**) Neutralization curves and half-maximal inhibitory concentration (IC_50_) values of the SD4, SD5, SD7, SD12, and SD22 against SFTSV MC16 (C3 genotype) and NB24 (J2 genotype) strains. (**E**) Similarity network of SFTSV-specific B cell clones. For each cell (node), the cases (color), number of CDR3 (diameter), and subtype of immunoglobulins (shape) are illustrated. Edges connect those clones with one amino acid substitution in the CDR3 region. Different colors correspond to different individuals. Different shapes indicate distinct subtypes of immunoglobulins. The diameter represents the number of CDR3 regions. (**F and G**) Neutralization curves of the SD4 family against the SFTSV MC16 and NB24 strains.

Twenty-one of the 22 antibodies could bind to Gn with OD_450_ measurements of >0.2 (cutoff), including 13 samples with OD_450_ values > 1. Meanwhile, all antibodies could also bind to SFTSV; however, only a single antibody (SD20) had an OD_450_ value of >1 (Fig. 2A). Furthermore, the correlation analyses revealed that there was no linear relationship between the binding activity against both Gn and SFTSV (Fig. S2). Neutralization experiments revealed five antibodies with potent neutralizing activities, including SD4, SD5, SD7, SD12, and SD22 (Table S2), which were selected for small-scale expression in HEK293F suspension cells and for validation of the ability to block SFTSV infection.

To accurately assess whether the screened antibodies possessed broad-spectrum activity against the major circulating SFTSV strains, we selected the prevalent C3 genotype in China and, also, the evolutionarily more distant J2 genotype circulating in South Korea to enable evaluation of the neutralizing breadth of the antibodies, including the previously-reported effective antibody SF5 employed as a control (15). These mAbs inhibited the SFTSV MC16 strain (C3 genotype) infection with varying potencies (IC_50_: 1.0–158.1 ng/mL). Similarly, these mAbs also inhibited the SFTSV NB24 strain (J2 genotype) infection, with variable potencies (IC_50_: 0.02–81.1 ng/mL; Fig. 2B through D). Of note, antibody SD4 exhibited the strongest neutralizing activity, with an IC_50_ of 1.0 ng/mL for MC16 and 0.02 ng/mL for the NB24 strain. In addition, the neutralization effects of SD22 and SD12 were also comparable. The IC_50_ values of SD22 were 1.1 ng/mL and 1.0 ng/mL for the SFTSV MC16 and NB24 strains, and those of SD12 were 2.6 ng/mL and 1.4 ng/mL, respectively (Fig. 2D).

Next, we screened additional antibodies with a single amino acid substitution in the CDR3 by constructing a clonal expansion mutation network (Fig. 2E) (24). The antibody SD4-S isolated from the same patient (P7) with SD4 and SD4-X from another recovered individual (P10) were also synthesized to test their neutralizing activities. Notably, the CDR3 region in the heavy chain of SD4-X only differed from that of SD4 at residue 108 (Y108F), whereas SD4-S differed from SD4 at positions 103 (Y103F) and 108 (Y108F); however, the CDR3 region in the light chain was more diverged between them (Fig. S3). We found that the IC_50_ values of SD4-S and SD4-X for MC16 were 0.8 ng/mL and 3.3 ng/mL, respectively (Fig. 2F), whereas those for NB24 were 0.4 ng/mL and 3.0 ng/mL, respectively (Fig. 2G).

### Binding characterization studies of the anti-SFTSV neutralizing antibodies

We used biolayer interferometry (BLI) to determine the binding of the seven human mAbs sorted above. Notably, SD4, SD4-S, and SD22 bound to SFTSV Gn (C3 genotype) at picomolar levels (Fig. S4). Remarkably, the *K_D_* of the highest-affinity antibody (SD4) was 96 pM to the SFTSV Gn glycoprotein (Fig. S4A through G), which was also consistent with the neutralization activity findings. However, the binding of antibody SD5 to SFTSV-Gn was not detected (data not shown). We then performed a competition-binding assay using BLI for representative mAbs to determine whether there are any overlapping antigenic sites between different mAbs. The results showed that SD4, SD4-S, SD4-X, SD12, SD22, and SF5 competed with each other with binding signals < 0.33 (Fig. S4H and I) (25). We also found that SD7 could still bind to Gn when used as the second antibody. Furthermore, mass spectrometry analysis revealed that the antigenic regions recognized by SD4 and SD12 were identical, with both located in the Y70-Y89 and C156-D170 regions (Fig. S5). However, apart from the aforementioned regions, SD7 could also recognize the L182-F191 region (Fig. S5). These findings suggested that the majority of these antibodies targeted common antigenic epitopes and antibody responses caused by natural SFTSV infection primarily focused on the N-terminal head region of Gn.

### Neutralizing antibodies recognized different genotypes of SFTSV

**Fig 3:**
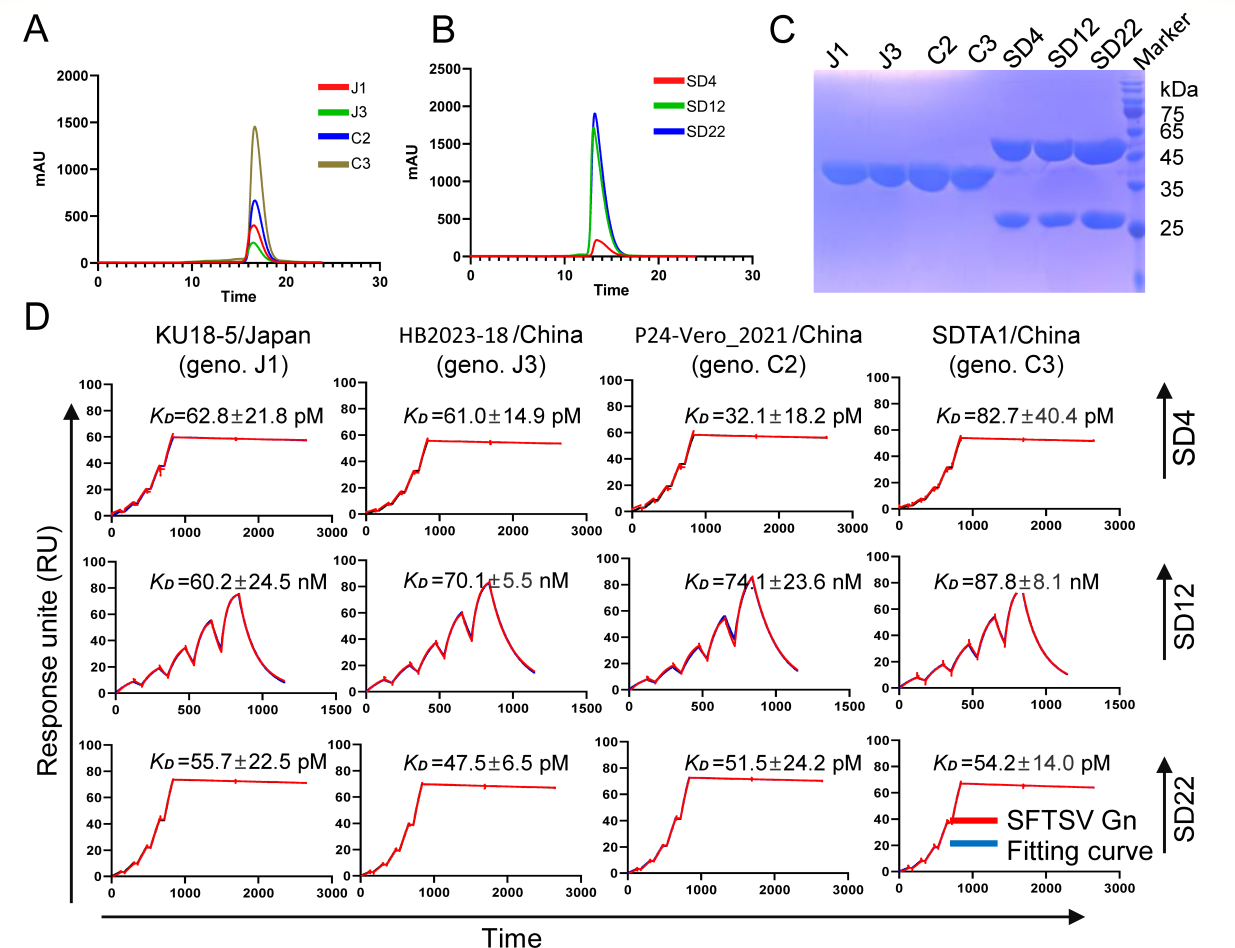
The characteristics of high-affinity cross-reactive human neutralizing antibodies to SFTSV-Gn of different genotypes. (**A and B**) Size-exclusion purification for the SFTSV Gn head (positions 19-337) of J1 (KU18-5/Japan), J3 (HB2023-18/China), C2 (P24-Vero_2021/China), and C3 (SDTA1/China) genotypes and the SD4, SD12, and SD22 antibodies. (**C**) The purity of the antigens and antibodies was determined by sodium dodecyl sulfate-polyacrylamide gel electrophoresis. (**D**) The surface plasmon resonance curves for the SD4, SD12, and SD22 mAbs bound to the Gn head of SFTSV genotypes J1, J3, C2, and C3. Raw and fitted curves are represented by red and blue lines, respectively. *K_D_* indicates mean ± SD from three independent repeats.

SFTSV has evolved into various co-circulating lineages, such as C2 and C3 genotypes prevalent in China, and J1 and J3 genotypes prevalent in Japan and South Korea (Fig. S6) (26, 27). Therefore, we determined the binding breadth of the three human mAbs (SD4, SD12, and SD22) to the major SFTSV genotypes (J1, J3, C2, and C3). To this end, we purified the antigens and antibodies (Fig. 3A through C) and tested their binding capacities employing surface plasmon resonance (SPR). The results showed that the mean *K_D_* values of SD4 with J1, J3, C2, and C3 were 62.8 pM, 61.0 pM, 32.1 pM, and 82.7 pM, whereas those of SD12 were 60.2 nM, 70.1 nM, 74.1 nM, and 87.8 nM and those of SD22 were 55.7 pM, 47.5 pM, 51.5 pM, and 54.2 pM, respectively (Fig. 3D). Importantly, statistical analyses showed that there were no significant differences in the binding affinities of these antibodies to different SFTSV genotypes (Fig. S7).

### SD4 and SD22 rescue A129 mice from lethal SFTSV challenge

To test the *in vivo* neutralizing activity of these antibodies, we subcutaneously injected type I interferon receptor-deficient (IFNα/βR^−/−^) 129 (A129) mice (*n* = 5 per group) with the SFTSV SDTA-1 strain (C3 genotype) at a lethal dose of 20 PFU. One day before the challenge, and 1 or 2 days after the challenge, we intraperitoneally injected the mice with either PBS or antibody SD4 with a single dose of 20 mg/kg of body weight. The mice in the 20 PFU SFTSV group began to die 7 dpi, and all mice died within 9 days, whereas all the mice treated with SD4 survived without any weight loss in the prophylactic and early mAb treatment groups (Fig. S8).

**Fig 4:**
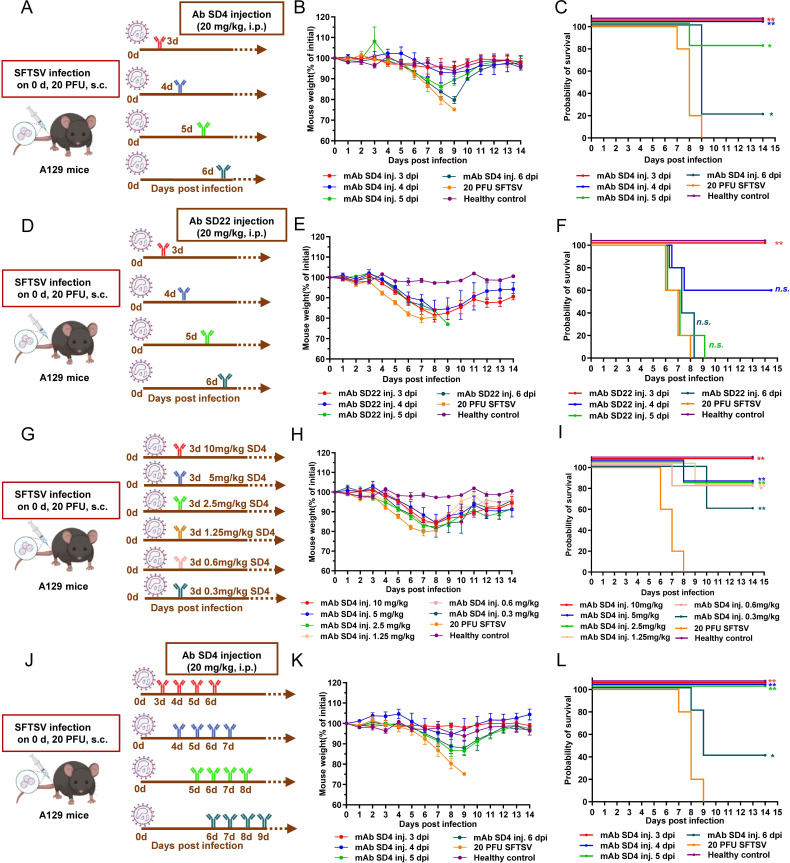
Delayed administration of SD4 and SD22 rescued mice from lethal SFTSV challenge. (**A**) The experimental design for SFTSV infection and the administration of a single dose of SD4 mAb. The type I interferon receptor-deficient (IFNα/βR^−/−^) 129 (A129) mice were infected with 20 PFU SDTA-1 strain (C3 genotype) of SFTSV through a subcutaneous route. The A129 mice were intraperitoneally treated with SD4 at 20 mg/kg body weight at 3, 4, 5, or 6 days post-inoculation (dpi). (**B and C**) Percentages of body weight change in the SD4 group compared with that on the day of virus inoculation and survival rates were monitored daily until 14 dpi. (**D**) The experimental design for SFTSV infection and the administration of a single dose of the SD22 mAb. (**E and F**) Body weight percentage change and survival rates were monitored daily until 14 dpi. (**G**) The experimental design for SFTSV infection and the administration of SD4 antibody with different treatment doses. A129 mice were administered the SD4 mAb at 10, 5, 2.5, 1.25, 0.6, and 0.3 mg/kg of body weight 3 dpi. (**H and I**) Percentages of body weight change and survival rates were monitored daily until 14 dpi. (**J**) The experimental design for SFTSV infection and the administration of SD4 antibody with four consecutive doses. A129 mice were infected with SFTSV as mentioned before. The mice were then intraperitoneally treated with SD4 at 20 mg/kg body weight at 3, 4, 5, or 6 dpi for 4 consecutive days. (**K and L**) Percentages of body weight change and survival rates with four consecutive doses were monitored daily until 14 dpi. All A129 mouse experiments used five animals per group. Male or female, weight-matched animals were randomly assigned and challenged subcutaneously with SFTSV, respectively. Healthy control refers to mice that were uninfected. Relative body weight change values were shown as the mean ± SEM in each group. The Kaplan-Meier method and log-rank test were used to analyze time-to-event data. *, *P* < 0.05; **, *P* < 0.01, ***, *P* < 0.001; ns, not significant. s.c., subcutaneous injection; i.p., intraperitoneal injection.

Clinically, most severe and critical SFTSV cases are diagnosed late. To better mimic this situation, we started to administer the SD4 or SD22 antibody treatment at 3, 4, 5, and 6 dpi with a single dose of 20 mg/kg per body weight (Fig. 4A through F). Though given in a delayed manner with weight loss, SD4 and SD22 protected all mice in the 3 dpi groups. Notably, SD4 also protected all mice in the 4 dpi group, 80% of the mice in the 5 dpi group (2% weight loss was observed in the surviving mice by day 14), and even 20% of the mice in the 6 dpi group (Fig. 4B and C). However, only 60% of the mice in the 4 dpi group survived when treated with SD22.

Since the SD4 antibody exhibited excellent protective effects *in vivo*, we further investigated whether it still possessed protective efficacy at lower dosages. A129 mice were infected with 20 PFU of the SFTSV SDTA-1 strain. SD4 was then intraperitoneally administered at 10, 5, 2.5, 1.2, 0.6, and 0.3 mg/kg body weight 3 dpi (Fig. 4G). The survival rates at 10, 5, 2.5, 1.2, 0.6, and 0.3 mg/kg body weight were 100%, 80%, 80%, 80%, 80%, and 60%, respectively, although the body weight decreased compared with the healthy control (Fig. 4H and I). Therefore, SD4 has potential as a therapeutic drug for clinical treatment even when administered at lower concentrations.

We next tested whether consecutive days of antibody treatment could provide greater clinical benefits in our SFTSV lethal challenge murine model. In this experiment, the antibody treatment started from 3, 4, 5, and 6 dpi and continued for 4 consecutive days (Fig. 4J). The mice with SD4 treatment 3, 4, and 5 dpi did not lose weight, whereas the mice in the 6 dpi group lost 3% of their body weight by day 14 (Fig. 4K). Similarly, all the mice in the 3 and 4 dpi groups survived. Notably, all the mice (100%) in the 5 dpi group and 40% of the mice in the 6 dpi group survived after multiple SD4 treatments (Fig. 4L). These results indicated that consecutive SD4 treatment could further enhance the survival rates of the mice in the 5 and 6 dpi groups.

### Structural basis of SD4, SD22, and SD12 binding to SFTSV-Gn

**Fig 5:**
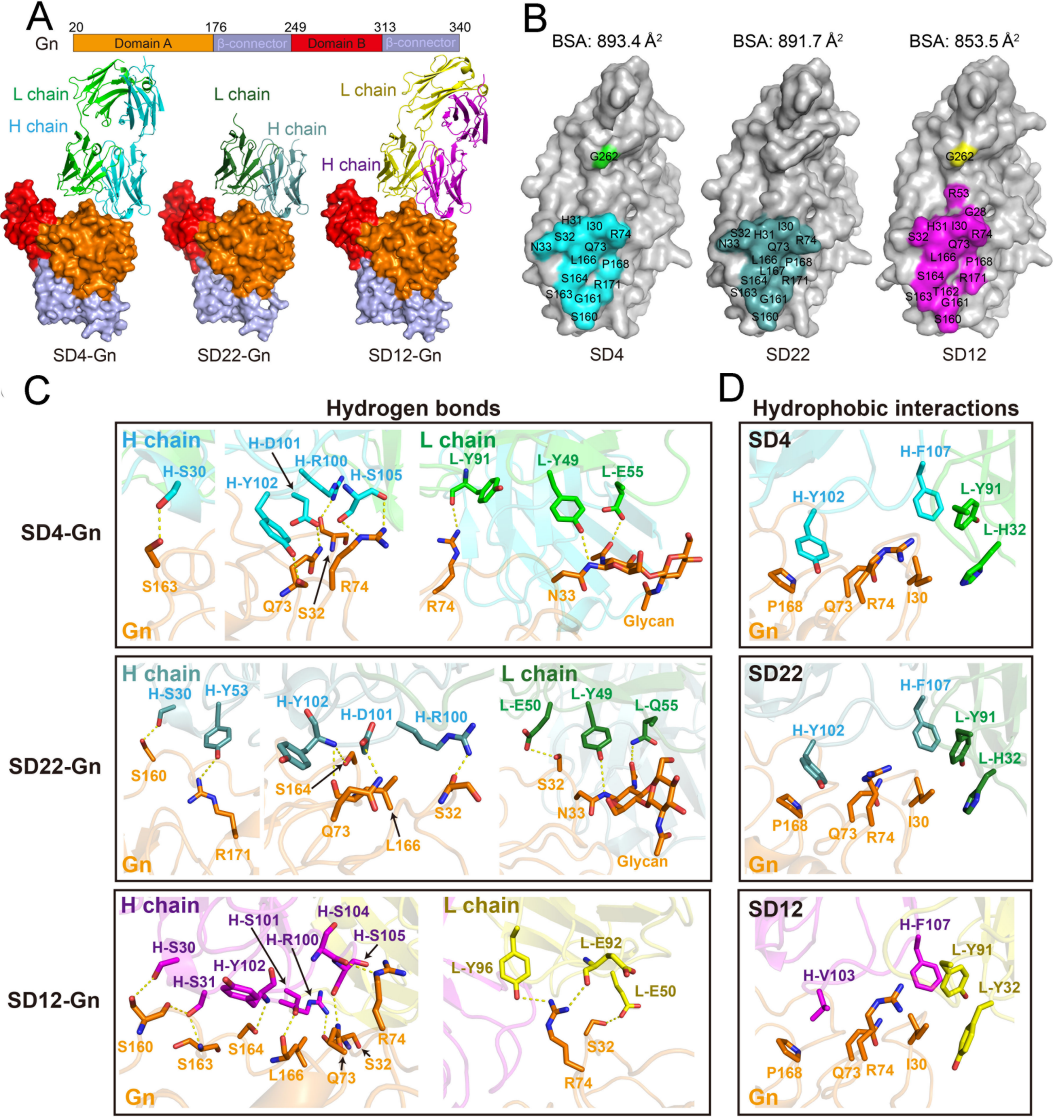
Structural analysis of SD4, SD22, and SD12 mAbs complexed with SFTSV-Gn proteins. (**A**) The overall structures of the Gn protein in complex with SD4 (left), SD22 (middle), and SD12 (right) mAbs are shown. The Gn protein is shown in surface form, with domain A, domain B, and the β-connector colored in orange, red, and light blue, respectively. The SD4, SD22, and SD12 mAbs are displayed in cartoon form, with the heavy chain in cyan, light teal, and magenta, respectively, and the light chain in green, forest, and yellow, respectively. (**B**) Surface representation of the contact residues on Gn interacting with SD4 (left), SD22 (middle), and SD12 (right). The binding residues in domain A interacting with SD4, SD22, and SD12 are highlighted in cyan, light teal, and magenta, respectively, whereas those in domain B are colored in green and yellow, respectively. The buried surface areas of the Gn-antibody interfaces are also shown. (**C and D**) Detailed views of hydrogen bonds (**C**) and hydrophobic interactions (**D**) between Gn and SD4 (top), SD22 (middle), and SD12 (bottom). Key interacting residues are depicted as stick structures and labeled. Hydrogen bonds, defined with a cutoff distance of 3.5 Å, are indicated by yellow lines.

To elucidate the molecular properties of SD4, SD22, and SD12 binding to the SFTSV Gn, we prepared protein complexes of SD4 Fab, SD22 Fab, and SD12 Fab with the Gn head and determined their structures using X-ray crystallography. The structures of the SD4-Gn, SD22-Gn, and SD12-Gn complexes were determined at 3.3 Å, 2.8 Å, and 2.4 Å, respectively. The Gn head consisted of three parties: domain A, domain B, and a β-connector (23). SD4, SD22, and SD12 bound to domain A at similarly-perpendicular angles (Fig. 5A). The buried surface areas (BSA) of Gn in complex with SD4 and SD22 were 893.4 Å^2^ and 891.7 Å^2^, respectively, which were slightly greater than that of SD12 (853.5 Å^2^; Fig. 5B). Sequence alignment analyses indicated that these structures shared a high degree of similarity, and the root mean square deviation (RMSD) values were 0.629 Å (508 Cα atoms) for SD22-Gn *vs*. SD12-Gn, 0.682 Å (497 Cα atoms) for SD4-Gn vs. SD22-Gn, and, finally, 0.959 Å (550 Cα atoms) for SD4-Gn vs. SD12-Gn (Fig. S9). Epitope mapping revealed that SD4, SD22, and SD12 largely recognized the same epitope, with only six of 18 residues differing among them, which was consistent with the findings from the competition-binding assays (Fig. 5B; see also Fig. S10).

Detailed interaction analyses revealed that both the heavy and light chains of SD4, SD22, and SD12 contributed to the antibody-Gn interaction, which was mediated by hydrogen bonding and hydrophobic interactions. Specifically, SD4 and SD22 formed eight hydrogen bonds with Gn and its associated glycan, whereas SD12 formed 11 hydrogen bonds (Fig. 5C). All three antibodies shared the key residues H-S30, H-R100, and H-Y102, which form hydrogen bonds with Gn residues S32, Q73, S160, S163, and S164. Additional shared interactions included H-D101 and L-Y49 in SD4 and SD22, which bind to Gn residues Q73 and N33. Pairwise overlaps were observed: H-S105 in SD4 and SD12 interacted with Gn residues S32 and Q73, whereas L-E50 in SD22 and SD12 bound to S32, Q73, and R74. Unique interactions were also identified for each antibody. Specifically, SD4 employed L-Y91, SD22 utilized H-Y53, and SD12 engaged multiple residues: H-S31, H-S101, H-S104, L-E92, and L-Y96, to interact with distinct Gn residues R74, S163, L166, and R171 (Fig. 5C).

Interestingly, SD4 L-E55 and SD22 L-Q55 formed a hydrogen bond with the N-linked glycan residue N33 of Gn, an interaction not observed in SD12 (Fig. 5C). Furthermore, residues H-F107, L-H32, and L-Y91 in SD4 and SD22 engaged in hydrophobic interactions with Gn residue I30, similar to SD12 residues H-F107, L-Y32, and L-Y91. However, SD4 and SD22 H-Y102 formed stronger hydrophobic interactions with P168 and the hydrophobic atom of Q73 and R74 side chains when compared with SD12 H-V103 (Fig. 5D), potentially accounting for the higher binding affinity of SD4 and SD22.

Moreover, a sequence comparison of the Gn protein recognized by SD4 antibodies in 1,618 SFTSV strains revealed that there were few amino acid substitutions at positions 30 (I30, 4.6%), 73 (Q73, 0.4%), and 161 (G161, 0.6%; Fig. S6). The remaining amino acids at the SD4-Gn interaction interface were highly conserved: 99.9%–100% at H31, S32, R74, S160, S163, S164, L166, P168, R171, and G264, which suggested the binding sites were extremely conserved among the prevalent SFTSV strains (Fig. S6).

### Neutralization mechanisms of mAbs SD4, SD22, and SD12

**Fig 6:**
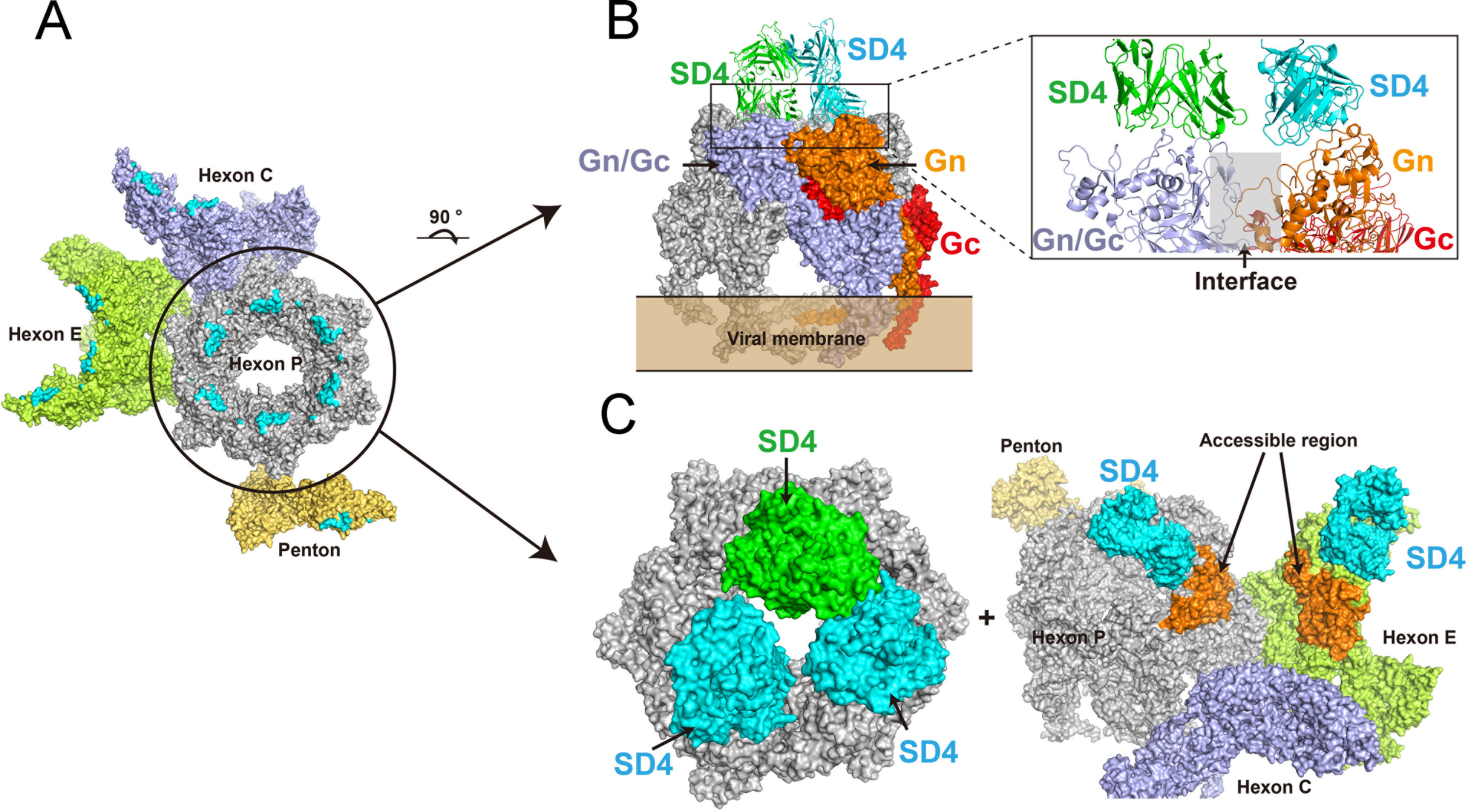
SD4 epitopes on the Gn protein in the SFTSV virion. (**A**) Epitope mapping on the SFTSV virion (PDB: 8I4T). The SD4 epitopes on the virion are highlighted in cyan. (**B**) Superimposed structure of two SD4-Gn complexes with a virion hexon unit. The Gn, Gc, and Gn/Gc regions are colored orange, red, and light blue, respectively. The SD4 mAbs are shown in green and cyan. (**C**) Top view of the superimposed structure of three SD4-Gn complexes bound to a virion hexon unit. Side view of the superimposed structure showing two SD4-Gn complexes bound to a hexon unit and an adjacent hexon unit of the virion. The SD4 antibodies are shown in green or cyan, with the accessible region highlighted in orange.

To investigate the neutralization mechanisms of SD4, SD22, and SD12, we superimposed the structures of the SD4-Gn, SD22-Gn, and SD12-Gn complexes onto the native SFTSV virion structure. Previous studies have shown that the SFTSV virion is composed of 12 pentons at the icosahedral vertices and 110 hexons covering the facets, yielding a total of 720 Gn/Gc heterodimers per virion (23, 28). Epitope mapping revealed that the SD4, SD22, and SD12 binding sites were located on the surfaces of the hexons or pentons (Fig. 6A; see also Fig. S11A through D). Upon binding to Gn, SD4, SD22, and SD12 did not disrupt the interface between protomers of Gn or the Gn-Gc interaction. Furthermore, no cross-binding to adjacent Gn proteins was observed for either antibody (Fig. 6B; see also Fig. S11B and E). These results suggest that SD4, SD22, and SD12 do not interfere with the Gn-Gc interaction or the assembly of Gn protein protomers.

Given the exposed positions of Gn on the top of the virion surface, we hypothesize that they facilitate interactions with host receptors and/or other proteins necessary for viral replication (29, 30). We observed that when three SD4 molecules bound to Gn within a hexon (Fig. 6C; see also Fig. S11C and F), they occupied much of the central region of the hexon, concealing more than half of the accessible Gn regions. This binding left only a small portion of the Gn exposed, specifically at the valley between two adjacent protomers (Fig. 6C; see also Fig. S11C through F), potentially preventing Gn interaction with host cell receptors.

### Structural comparison of SD4 with previously reported Gn-binding antibodies SF5 and S2A5

Two previously reported neutralizing antibodies, SF5 and S2A5, which bind to domain A of the Gn head (15, 16), were selected as controls, and we next compared the complex structures of SD4 with those of SF5 and S2A5. Structural comparison revealed that SD4 partially overlapped in its binding region with SF5, but not with S2A5 (Fig. S12A). Detailed binding analysis showed that the BSA of SD4 was smaller than that of SF5 (893.4 Å² vs 1188.5 Å²), but comparable with that of S2A5 (893.4 Å² vs 861.0 Å²) (Fig. S12B). Analysis of the binding residues revealed that five of the 27 residues involved in SF5 binding overlapped with those of SD4, whereas there was no overlap between the binding residues of SD4 and S2A5 (Fig. S12B).

We next docked the structures of these two antibodies onto the virion. Unlike SD4, SF5 was found to bind to the inner surface of the hexon. Like SD4, SF5 exhibited steric clashes with neighboring Gn-bound SF5 molecules, although it did not interfere with the interaction between adjacent Gn molecules (Fig. S12C). These clashes may reduce the accessibility of SF5 to the Gn surface at the top of the virion, thereby contributing to its lower neutralizing activity compared with SD4. In contrast, S2A5 bound to the outer surface of the hexon; however, its interaction with Gn was hindered by an adjacent Gn molecule (Fig. S12D), suggesting that its neutralization mechanism was distinct from that of SD4.

## DISCUSSION

Conceptually, mAb therapy is a potentially safe and effective therapeutic approach against SFTS, and many mAb drugs have been approved for Ebola, HIV, and human respiratory syncytial virus (31). Due to the increasing SFTS disease incidence, antivirals against SFTS are urgently needed in China and elsewhere in Asia. In this study, we used the SFTSV-Gn glycoprotein as baits to fish binding B cells from convalescent individuals by scRNA-seq and further validated the potential efficacy of these antibodies (32). The screened antibodies not only possessed high binding affinities but also broad-spectrum neutralizing activities against the different SFTSV strains in circulation. Importantly, the therapeutic experiments in the mouse model have demonstrated that the SD4 antibody could effectively rescue moribund mice following challenge with lethal SFTSV infection. Mechanistically, the antibodies SD4, SD22, and SD12 targeted the Gn head at domain A and prevent virus binding to host cells.

To improve the efficiency of the discovery of neutralizing mAbs, the transcriptome and immunoglobulin repertoire of Gn-specific B cells that produce neutralizing antibodies were explored. As expected, the neutralizing mAbs SD4, SD5, and SD12 were derived from plasma cells, and SD7 was derived from transitional B cells; however, SD22, screened by enriched clonotypes, belonged to IgG subtype B cells (33). We observed that the CDR3 of SD4, SD5, SD7, and SD12 antibodies was identical to the germline genes (Table S1), suggestive of an absence of somatic hypermutation. Furthermore, the antibodies SD4 and SD22 with the highest affinity were of the germline IGHV3-30 genotype, which was consistent with the previously reported antibody Ab10 (19). However, the neutralizing antibodies SF1, SF64, and SF71 belonged to the IGHV5-51 genotype, which we noted was one of the top five genotypes in our study (15); therefore, we speculate that those specific germline genes may exert an important role in affecting SFTSV clearance.

As shown in Fig. 1A, the SFTS inpatients are predominantly elderly (interquartile range [IQR]: 54–70 years) (14). The antibody diversity in the convalescent donors may indeed be somewhat limited due to their elderly age and immune senescence. Indeed, a pervasive age-dependent loss of immune repertoire diversity has been reported, as evidenced in studies of human peripheral blood and model organisms (34, 35). Therefore, immunological deterioration may contribute to the higher mortality rates observed among elderly SFTS patients.

Neutralizing activity is of vital importance for an antibody drug. Of the five antibodies identified here, the SD4 antibody family (SD4, SD4-S, and SD4-X), SD12, and SD22 each possessed IC_50_ values of <4 ng/mL. Among them, the IC_50_ values of SD4, SD4-S, and SD22 were even <1 ng/mL to both the diverged C3 and J2 genotypes of SFTSV-Gn. Considering the high sequence conservation of the amino acids in the Gn-antibody interaction interface, we believe that SD4 and SD22 possess broad-spectrum neutralizing activity and are also able to neutralize other genotypes of SFTSV with high efficacy. In particular, these human-derived antibodies also possessed strong and broadly binding affinities against SFTSV-Gn derived from distinct viral genotypes. The *K_D_* values of the antibody SD4 ranged from 32.1 to 82.7 pM for the different genotypes of SFTSV, and those of SD22 were also <60 pM, which is comparable with the previously described JK-2 (*K_D_* = 21.2 pM) and JK-8 (*K_D_* = 1 pM) (21). Notably, previously reported Gn-specific mAbs such as SF5 (*K_D_* = 339.3 nM), SF71 (*K_D_* = 476.8 nM), SNB02 (*K_D_* = 5.5 nM), B1G11 (*K_D_* = 0.19 nM), and Ab10 (*K_D_* = 0.104 nM) were approximately 3-fold to 100-fold higher than SD4 and SD22 identified in the present work (15, 16, 18, 19).

SD12 exhibited a lower binding affinity and reduced neutralizing potency compared with SD4, suggesting a positive correlation between binding affinity and neutralizing efficacy. Structural analyses also revealed that SD12 formed more hydrogen bonds; however, it engaged in weaker hydrophobic interactions with Gn than SD4. This weaker interaction may be attributed to H-V103, suggesting that a V103Y mutation could potentially enhance binding affinity and neutralizing ability of SD12, which warrants further investigation.

Prior reports have shown that the SFTSV viral loads in fatal cases were significantly higher than those of the surviving patients (36). Therefore, timely diagnosis of SFTSV and antibody treatment are likely key to improving survival rates. In murine models, after injecting a lethal dose of SFTSV, a single dose of each mAb (i.e. 10 mg/kg SF5, 3 dpi 100% survival and S2A5 with 400 µg/mouse, 2 dpi 33% survival), and four doses of mAb (SNB02 400 µg/mice, 1 dpi 100% survival and Ab10 600 µg/mouse, 3 dpi 80% survival) were tested for protective efficacy. It is important to note that the majority of hospitalized SFTS cases are diagnosed late with correspondingly higher fatality rates; however, none of the antibodies reported previously in the literature have been demonstrated to be able to effectively rescue severe and critical SFTSV cases (37). Herein, we show that a single dose of SD4 at 20 mg/kg animal weight could provide 100%, 80%, and 20% protection for the mice in the 4, 5, and 6 dpi groups. Importantly, a lower dosage of 0.3 mg/kg of SD4 afforded up to 60% protection when treated 3 dpi. Notably, when given four doses of SD4, the survival rates increased to 100% in the 5 dpi group and 40% in the 6 dpi group. These results indicated that the SD4 has excellent potential for effective treatment of hospitalized, severe, and critical SFTS patients.

The present study has several limitations. To reduce confounding effects from blood collection, we only evaluated the efficacy of the antibodies by monitoring body weight changes and mortality post-infection. We will examine the viral load kinetics and histopathological alterations in mouse tissues after antibody treatments in the future. Additionally, there was no notable difference in the body weight changes measured between the single and consecutive treatment groups, and the survival difference is limited to a single mouse. Hence, increasing the number of mice per group in further work will offer insights into any potential survival benefits in the consecutive treatment group.

In summary, we have identified and characterized >20 SFTSV-Gn mAbs from recovered SFTSV cases. Seven mAbs targeting SFTSV Gn glycoproteins were functionally validated. Among them, SD4, SD22, and SD4-S bound to SFTSV-Gn at pM levels and also showed excellent neutralization efficacies (<1.1 ng/mL) against divergent genotypes of SFTSV in vitro. Most significantly, SD4 could rescue 80% of the mice with a lethal challenge of SFTSV when given at 5 dpi, and consecutive days of SD4 treatment could further enhance the survival rates. Furthermore, the crystallographic structures of SD4, SD22, and SD12 with the Gn antigen were determined, and the three antibodies bound to Gn within the hexon region, which we hypothesize prevents interactions with host cell receptors required for infection. Overall, we have described several promising mAbs against SFTSV, with considerable potential for clinical treatment of severe and critical SFTS cases. This knowledge will provide a basis for rational antibody vaccine design against SFTSV to mitigate the morbidity and mortality caused by this emerging high-consequence pathogen.

## MATERIALS AND METHODS

### Cells, animals, and blood sample processing

Vero cells were purchased from the Cell Resource Center, Institute of Basic Medical Sciences, and maintained in Dulbecco’s modified Eagle medium (DMEM) containing 10% fetal bovine serum and incubated at 37°C under 5% CO_2_. The SFTSV SDTA-1 strain was propagated and titered in Vero cells. The cell supernatants were harvested at day 5 post-infection and stored at −80°C as 1 mL aliquots. The suspension cell passage of HEK293F was maintained in SMM293-TII medium supplied by Sino Biological Inc. (Beijing, China) on a shaker at 37°C under 5% CO_2_. A129 mice were maintained at the Key Laboratory of Etiology and Epidemiology of Emerging Infectious Diseases in the Universities of Shandong, Shandong First Medical University (9). The male or female mice with weights from 20 to 32 g were used for virus challenge experiments. Blood from each convalescent SFTS patient was processed employing human lymphocyte separation tubes (Dakewe) to isolate PBMCs, according to the manufacturer’s instructions. These cells were cryopreserved in liquid nitrogen before analysis. The plasma was separated and stored at −80°C until further use (38).

### Cell staining and flow cytometry

The recovered PBMCs were stained with SFTSV-Gn antibodies according to the previous description (38). Briefly, cells were labeled with 400 nmol of SFTSV-Gn for 30 min at 4°C, after which the cells were washed twice following centrifugation (450 × *g* for 5 min) and resuspended in FACS Buffer, that is, 2% fetal bovine serum (FBS) in phosphate-buffered saline (PBS). Cells from different subjects were then stained with different TotalSeq C Abs: 1 (TotalSeq-C0251), 2 (TotalSeq-C0253), 3 (TotalSeq-C0255), 4 (TotalSeq-C0257), and 5 (TotalSeq-C0259). Cells were simultaneously stained with PE mouse anti-human CD45 Ab, FITC mouse anti-human CD3 Ab and CD16 Ab, BV421 mouse anti-human CD27 Ab, PerCP/Cyanine5.5 mouse anti-human IgG Ab, and APC anti-His Ab and incubated for 30 mins at 4°C before washing and resuspension in PBS supplemented with 5% FBS. Cells were then stained with A700 for 15 min at 4°C and washed and resuspended in PBS containing 4% FBS. Antigen-binding B cells were then sorted for CD45^+^CD3^-^CD16^-^ SFTSV-Gn^+^ cells using a BD Aria III cell sorter with FACS modules. After sorting, 10 separate samples were combined and concentrated cells and counted using a hemocytometer and microscopy, before resuspending up to 10,000 cells in a volume of 32 µL for 5' single-cell RNA-seq.

### Droplet-based single-cell RNA sequencing

Single-cell capturing and library construction were performed using the Chromium Next GEM Single-Cell V(D)J Reagent Kits v2 (10 × Genomics), according to the manufacturer’s instructions. The cell suspension (living cells determined by Count Star > 85%), barcoded gel beads, and partitioning oil were loaded onto the Chromium Chip to generate single-cell Gel Beads-in-Emulsion (GEMs). Approximately 8,000 cells were added to each channel, and approximately 4,000 target cells were recovered. Captured cells were lysed, and the transcripts were barcoded through reverse transcription inside individual GEMs. Then cDNA, along with cell barcodes, was PCR-amplified at 53°C for 45 min, followed by 85°C for 5 min, and a hold at 4°C. The scRNA-seq libraries were constructed by using the 5' library kits, and the scBCR-seq libraries were constructed by using the V(D)J Enrichment Kits. The constructed libraries were sequenced on an Illumina NovaSeq 6000 platform to generate 2 × 150 bp paired-end reads (39–41).

### Recombinant protein expression and purification

The following criteria were used to identify neutralizing mAbs based on previous studies (24, 33). First, B cell clonotypes with frequencies greater than one in each repertoire; second, the IgG1 and IgG3 subtype was prioritized for further verification; third, the top-ranked antibodies, based on the UMI number, were synthesized; fourth, the subjects with the high serum neutralization titers were prioritized as ideal candidates; and fifth, naïve and resting B cells were excluded for antibody synthesis. Finally, mAbs lacking transcriptional data were omitted to ensure “high-confidence” antigen specificity.

The Gn proteins used for bait, ELISA, SPR, and BLI experiments were expressed using HEK293F cells (Sino Biological). The coding sequence for Gn glycoproteins (GenBank: AQS99580, G19-K452, C3 genotype) was inserted into pcDNA3.4 vectors, incorporating a C-terminal 6 × His tag. Transient transfection of HEK293T cells was performed, and the supernatants containing the expressed proteins were harvested. The supernatants were centrifuged at 12,000 rpm for 30 min to remove cellular debris. The Gn proteins were then purified using affinity chromatography on a HisTrap HP 5 mL column (Cytiva). Elution was carried out with a buffer consisting of 20 mM Tris (pH 8.0), 150 mM NaCl, and 300 mM imidazole.

Antibody VDJ heavy chain and paired VJ light chain sequences of selected mAbs were cloned into pcDNA3.4 expression vectors with IgG1 heavy chain, kappa light chain, or lambda light chain constant regions and amplified in *E. coli* DH5α. These plasmids were co-transfected into Expi293F cells, following the manufacturer’s instructions (Sina Biological). Cell cultures were harvested after 5 days of incubation at 37°C with shaking. Culture containing antibodies was frozen at −80°C until use. The thawed culture was centrifuged and passed through a HiTrap Protein A FF (GenScript) column. Antibodies were eluted using 0.1 M glycine (pH 3.0) and further purified by size-exclusion chromatography on a Superdex 200 10/300 Gl column (Cytiva). Detailed information is provided in the supplemental material.

### SFTSV neutralization assay

The neutralizing ability of monoclonal antibodies was further assessed using the 50% reporter signal reduction neutralization test (NT_50_) with SFTSV-mScarlet (GenBank: KR698337, J2 genotype; GenBank: KY440776, C3 genotype). Vero cells (15,000 cells per well in medium containing 2% FBS) were seeded into 96-well clear-bottom black polystyrene microplates (Corning). At 16 h post-seeding, 60  µL of 2-fold serially diluted mAbs were mixed with 60  µL of SFTSV-mScarlet (MOI = 0.01) and incubated at 37°C for 1 h. Subsequently, 100  µL of the virus-antibody complexes were transferred to each well of a 96-well plate. After 48 h of incubation at 37°C under 5% CO_2_, red fluorescence-positive cells were visualized and quantified using the EVOS imaging system (Invitrogen). The relative red fluorescence signal was calculated by normalizing the fluorescence of antibody-treated groups to that of no-antibody controls. NT_50_ values were estimated using a four-parameter logistic regression model with GraphPad Prism 8.0 software. Further information is provided in the supplemental material.

### Enzyme-linked immunosorbent assay for SFTSV-Gn-specific antibodies

Antigens were coated on BIOFIL high-binding 96-well plates at a concentration of 1 µg/mL SFTSV-Gn or approximately 10^5^ TCID50 SFTSV with 150 µL/well in 1:20 dilution in coating buffer (pH 9.6) overnight at 4°C. Plates were then washed three times with 200 µL/well of wash buffer (0.05% Tween-20 in PBS buffer). Plates were blocked by adding 150 µL/well of blocking buffer (5% milk in PBS) for 1 h at 37°C. Plates were then washed as described above; 100 µL of diluted serum samples in dilution buffer (3% BSA in PBST) or mAb binding was assessed at starting concentrations of 0.2 mg/mL for IgG were added to the wells and incubated for 30–60 min at 37°C. Plates were then washed five times as described above; 100 µL/well of 1:4,000 diluted Ab detection solution (Goat Anti-Human IgG/HRP, Solarbio) was added to the wells and incubated for 60 min at 37°C. Plates were then washed five times as described above; 60 µL/well of 3,3′,5′,5′-tetramethylbenzidine peroxidase substrate (Multi science) was then added to the wells and incubated at 37°C for 10 min. The reaction was then stopped by adding 60 µL/well of stop solution (0.5 M H_2_SO_4_) to each well. Absorbance at 450 nm was read and recorded. A bar chart employing the normalization data derived by subtracting background absorbance was generated using GraphPad Prism 8.0.

### Binding affinity and binding competition assays

For the BLI assay, the antibody binding screening and the competitive binding of mAbs and the SFTSV-Gn protein (or between two antibodies; GenBank: AQS99580, C3 genotype) were measured by BLI using the Octet RED96 system (FortéBio). All experiments were performed at room temperature, and the biosensors were pre-equilibrated in a buffer containing PBS with 0.005% (vol/vol) Tween-20 (PBST) for 10 min. To determine the competitive characteristics, 50 µg/mL of Gn was loaded onto biosensors for 60 s and flowed with 150 nM of the first protein (one antibody) for 240 s and the second protein for 240 s. The interference patterns from the Gn with buffer and the uncoated biosensors with protein were analyzed as two sets of controls. We used corrected data to compare the competitive characteristics by Octet data analysis software.

For the SPR assay, the SFTSV-Gn antigens (GenBank: LC473505, J1 genotype; HB2023-18, J3 genotype; GenBank: OP379669, C2 genotype; GenBank: AQS99580, C3 genotype) and antibodies were diluted in PBST buffer, and 10 nM of antibodies were immobilized on protein A chip (Cytiva). Gradient concentrations of SD12 from 200 nM to 12.5 nM, and SD4 as well as SD22 from 10 nM to 0.625 nM with 2-fold dilutions were flowed over the chip in PBST buffer. Binding affinities were measured using a BIAcore 8K (Cytiva) at 25°C in the single-cycle mode. Binding kinetics were analyzed with BIAcore Insight software using a 1:1 binding model. The Protein A chip was regenerated using 10 mM Glycine-HCl (pH 1.5). The values indicate the mean ± SD of three independent experiments.

### Animal experiments

We did not use any statistical methods to predetermine sample sizes for the animal studies. All IFNα/βR^-/-^ gene-deficient A129 mouse experiments used five animals per group (9). Male or female, weight-matched animals were randomly assigned and challenged subcutaneously with 20 PFU of SFTSV (GenBank: AQS99580, C3 genotype), respectively. For virus dilution and infection, the harvested SFTSV (4 × 10^6^ PFU/mL) was diluted with PBS to a final concentration of 200 PFU/mL, and then, 100 µL of the diluted virus was subcutaneously injected into the inguinal folds of the mice. For individual dose antibody inoculation experiments, a single dose of monoclonal antibody (20 mg/kg) or PBS (marked as 20 PFU SFTSV, *n* = 5) was intraperitoneally administered at different time points before or post-infection. For multiple-dose antibody inoculation experiments, mAbs (20 mg/kg) or PBS (marked as 20 PFU SFTSV, *n* = 5) were intraperitoneally administered to mice at different time points up to 4 days. For antibody treatment experiments at different concentrations, 10, 5, 2.5, 1.2, 0.6, and 0.3 mg/kg of mAbs were intraperitoneally administered at 3 dpi. Animals were weighed daily post-infection. Mice were monitored daily for survival for 14 days post-infection. Mice exhibiting weight loss of over 25% of their initial weight on day 0 were euthanized (42, 43).

### Complex preparation and crystallization

The sitting-drop vapor diffusion method was used to obtain high-resolution crystals of the Fab-Gn complexes. Purified SD4 Fab, SD22 Fab, or SD12 Fab was mixed with Gn protein and incubated on ice overnight. Following incubation, the complexes were further purified using Superdex 200 Increase 10/300 Gl columns (Cytiva) in a buffer containing 20 mM Tris (pH 8.0) and 50 mM NaCl. The complex proteins were concentrated to 5 and 10 mg/mL. Then, 1 µL of protein was mixed with 1 µL of reservoir solution at 18°C. High-quality SD4 Fab-Gn complex crystals were grown in 0.2 M ammonium acetate, 0.1 M sodium acetate trihydrate pH 4.6, 30% wt/vol polyethylene glycol 4,000. SD22 Fab-Gn crystals were observed in a solution of 4% (+/−)−2-methyl-2,4-pentanediol, 0.1 M citric acid pH 3.5, and 20% polyethylene glycol 1,500. SD12 Fab-Gn crystals were obtained using a solution of 0.1 M magnesium chloride hexahydrate, 0.1 M sodium HEPES, pH 7.0, 15% wt/vol PEG 4000.

### Model building and structure refinement

To collect X-ray diffraction data, all crystals were flash-cooled in liquid nitrogen after a brief incubation in their respective reservoir solutions, supplemented with 20% (vol/vol) glycerol as a cryoprotectant. X-ray diffraction data for the SD4-Gn, SD22-Gn, and SD12-Gn complexes were collected at the Shanghai Synchrotron Radiation Facility (SSRF), beamline BL02U1. The diffraction data were indexed, integrated, and scaled using HKL2000 software (44). Molecular replacement was performed using Phaser, with previously-determined structures of Gn (PDB: 5Y10), SD4 Fab (PDB: 7L2E), SD22 Fab (PDB: 7L2E), and SD12 Fab (PDB: 6UTA) serving as search models. Atomic models were built using Coot, and structure refinement was carried out with Phenix.refine (45, 46). The stereochemical quality of the final models was evaluated using MolProbity (47). Data collection, processing, and refinement statistics are provided in Table S3. All structural figures were generated using PyMOL (https://pymol.org/2/).

### Phylogenetic analysis

The complete genome sequences of the SFTSV M genes were obtained from GenBank on September 24, 2024, and aligned using MAFFT v7.490. Phylogenetic analysis was conducted with RAxML v8.1.6, applying the GTRGAMMA nucleotide substitution model and 1,000 bootstrap replicates (48). The resulting trees were visualized using FigTree v1.4.3, with M gene clades of SFTSV defined based on previous studies (27).

### Quantification and statistical analysis

#### scRNA-seq analysis

FastQC software was used for assessment of the quality of the raw sequencing data. The raw sequence reads were projected onto the human reference version GRCh38 (GRCh38, https://cf.10xgenomics.com/supp/cell-exp/refdata-gex-GRCh38-2020-A.tar.gz) using CellRanger 3.0.2 software pipeline. The output filtered gene expression matrices were analyzed by R software (v.4.0) combined with the Seurat package (v.3.0.0). Cells with high-quality transcriptomic information were kept for the downstream analysis by filtering out the cells with <300 detected genes or UMI numbers from 800 to 16,000 (49). The *ScaleData, DimPlot, RunPCA, FindNeighbors*, and *FindAllMarkers* functions were used to identify the true dimensionality of the data sets, as recommended by the Seurat developers. All details regarding the Seurat analyses performed in this work can be found on the website tutorial (https://satijalab.org/seurat/articles/seurat5_sketch_analysis).

#### B-cell type annotation and analysis

The dominant B and plasma cells were extracted from the data sets. Next, these major cell types were integrated for further subclustering. Scaling, principal component analysis, and clustering were further analyzed, as described above (49). All cells underwent dimensionality reduction and were subclustered together in a two-dimensional space according to common features. The function *FindAllMarkers* in Seurat was used to identify the 50 most highly-expressed genes in each cluster of cells, providing a comprehensive understanding of cell types based on the top genes and classic biomarkers reported in the literature (50).

Cell Ranger (v.3.0.2) vdj pipeline with GRCh38 as reference was used for BCR clonotype assignment while generating the assembled V(D)J sequences (https://www.10xgenomics.com/support/universal-three-prime-gene-expression). Nevertheless, only the cells with high-quality V(D)J contig sequences were selected, and V(D)J gene annotations were assigned by using IGBLAST software with the Change-O R package. A clonotype was defined based on each unique complementarity-determining region 3 (CDR3) amino acid sequence.

#### Statistical analysis

All statistical analyses were performed using GraphPad Prism 8 software (GraphPad Software). Data were presented as means ± standard deviation of experiments in triplicate. The two-tailed Student’s *t* test was utilized to analyze the difference between two groups, and the difference in three or more groups was analyzed by Brown-Forsythe ANOVA tests with Holm-Sidak’s multiple comparisons test. The Kaplan–Meier method and log-rank test were used to analyze time-to-event data. A non-linear model was selected for fitting analysis. Differences with *P* < 0.05 (*), *P* < 0.01 (**), or *P* < 0.001 (***) were considered statistically significant.

## Data Availability

Raw scRNA-seq files were deposited into the GSA database under accession number HRA009044 (https://www.cncb.ac.cn/). The accession numbers for the atomic coordinates and diffraction data reported in this paper are PDB 9JQU (crystal structure of SFTSV-Gn and SD4 antibody complex) and 9JQV (crystal structure of SFTSV-Gn and SD12 antibody complex). Source data are provided with this paper. Further data that support the ﬁndings of this study, including source codes of the scRNA-seq and Ab repertoire analyses, are available from the corresponding author upon reasonable request.

## SUPPLEMENTAL MATERIAL

Supplemental material jvi.00533-25-s0001.pdf Supplemental text, Tables S1 to S3, and Fig. S1 to S12.

ASM does not own the copyrights to Supplemental Material that may be linked to, or accessed through, an article. The authors have granted ASM a non-exclusive, world-wide license to publish the Supplemental Material files. Please contact the corresponding author directly for reuse.
