# Supplementary material for: Molecular mechanism of potently neutralizing human monoclonal antibodies against severe fever with thrombocytopenia virus infection
Source: J Virol. 2025 Jun 20;99(7):e00533-25. doi: 10.1128/jvi.00533-25 (PMC12282080; doi:10.1128/jvi.00533-25)
Supplement: Supplemental material — Supplemental text, Tables S1 to S3, and Fig. S1 to S12. [file jvi.00533-25-s0001.pdf]

Molecular mechanism of potently neutralizing human monoclonal antibodies against severe fever with thrombocytopenia virus infection

Chuansong Quan, Kaixiao Nie, Dezhen Ma, Chao Su, Lianfeng Li, Wenjun Zheng, Chunhong Yin, Yiwen Wang, Peipei Yang, Dingkun Peng, Xin Liu, Weiwei Li, Weixiao Liu, Chao Shan, Jie Zheng, Di Liu, Hong Zhang, Michael J. Carr, George F. Gao, Jianxun Qi, and Weifeng Shi

Content

1. Protein expression and purification..4

2. SFTSV neutralization assay 4

Table S1. Germline analysis of the potential mAbs targeting SFTSV Gn.................6

Table S2. The neutralization efficacy test of the expressed antibodies using qRT-PCR. 9

Table S3. Crystallographic data collection and refinement statistics.......................10

Fig. S1 The study design and single-cell transcriptional profiling of SFTSV-Gn specific B cells from recovered patients. 13

Fig. S2 The correlation of expressed mAbs against SFTSV-Gn protein and SFTSV. 14

Fig. S3 The sequence alignment of SD4 derived antibodies including both heavy and light chains..15

Fig. S4 Binding affinity analysis of the interaction between the mAbs and the SFTSV-Gn glycoprotein. 16

Fig. S5 The epitopes of the neutralizing mAbs were determined by mass spectrometry. 18

Fig. S6 Phylogenetic analyses of the M gene sequences of SFTSV. 19

Fig. S7 The binding ability of different neutralizing antibodies to SFTSV-Gn of different genotypes. 20

Fig. S8 Protective efficacy of SD4 in type I interferon receptor-deficient (IFNα/βR$^{-/-}$) A129 mice. 21

Fig. S9 Structural alignment of SD4-Gn, SD22-Gn, and SD12-Gn complexes. 22

Fig. S10 Protein sequence alignment of the SFTSV-Gn head from of four genotypes 23

Fig. S11 SD22 and SD12 epitopes on the Gn protein in the SFTSV virion. 24

FIG. S12 Binding of SD4, SF5, and S2A5 to the Gn protein in the SFTSV virion. 26

1. Protein expression and purification

For crystallization studies, the head domain of Gn (GenBank: AQS99580, G19-L337, C3 genotype) was re-engineered to enhance protein expression. This construct was transiently transfected into HEK293F cells, and the secreted proteins were purified using HisTrap HP columns, followed by size-exclusion chromatography on a HiLoad 16/60 Superdex 200 pg column (Cytiva) in a buffer containing 20 mM Tris, 150 mM and NaCl, pH 8.0.

For the crystallization experiments, purified SD4, SD22, and SD12 antibodies were digested with the immobilized papain (Thermo Scientific) according to the manufacturer's instructions. The monoclonal antibodies were pre-equilibrated in a buffer of 20 mM Na_3PO_4, 10 mM EDTA (pH 7.0). Papain digestion was conducted overnight at 37°C in the same buffer supplemented with 10 μM cysteine-HCl. The Fab fragments were purified by HiTrap Protein A FF (Cytiva) and size-exclusion chromatography (HiLoad 16/60 Superdex 200 pg column).

2. SFTSV neutralization assay

For neutralization, Vero cells in DMEM supplemented with 10% FBS, and 1% penicillin-streptomycin were seeded at 40,000 cells per well into clear-bottom white-walled 48-well plates and cultured overnight at 37°C. Equal volumes of heat-inactivated serum or plasma, and virus (GenBank: AQS99580, C3 genotype) diluted to 100 50% tissue culture infectious dose ($TCID_{50}$) were added together and incubated at 37°C for 1 h. The 400 μL mixture was transferred to a confluent layer of Vero cells and incubated

for 3 h, and washed three times, and cultured at 37°C for 96 h under 5% CO_2. The results were directly determined by the SYBR-qPCR assay, and the cut-off value was ≤30, or by IFA. Serum samples were tested with a starting dilution of 1:20, followed by two-fold serial dilutions to 1:640. The neutralization titers were calculated as the reciprocal of the serum dilution. In each assay, negative and positive control samples and virus back titrations were utilized to confirm the testing system stability. Experiments were repeated independently for rigor and reproducibility.

Table S1 Germline analysis of the potential mAbs targeting SFTSV Gn.

mAb	Patient	Subtype	Identity of V gene (%)	Identity of CDR3 (%)	Chain	V_gene	D_gene	J_gene	C_gene	Reads[a]	UMIS
SD1	P4	Plasma	91.9	89.5	IGK	IGKV3-15		IGKJ2	IGKC	56898	8775
SD1	P4	Plasma	81.8	100	IGH	IGHV1-69D		IGHJ3	IGHG1	20624	3842
SD2	P7	Transitional	96.6	88	IGL	IGLV1-40		IGLJ2	IGLC2	49230	1078
SD2	P7	Transitional	96.2	85.7	IGH	IGHV4-59		IGHJ4	IGHG1	25288	621
SD3	UN	Plasma	98.6	94.7	IGK	IGKV1-5		IGKJ1	IGKC	58044	847
SD3	UN	Plasma	96.2	100	IGH	IGHV3-13		IGHJ3	IGHG1	17870	334
SD4	P7	Plasma	98	100	IGH	IGHV3-30		IGHJ4	IGHG1	10978	279
SD4	P7	Plasma	96.5	95.5	IGK	IGKV1-33		IGKJ3	IGKC	66470	1492
SD5	P7	Plasma	92.6	100	IGH	IGHV3-64D		IGHJ4	IGHG1	12588	154
SD5	P7	Plasma	94.3	78.6	IGK	IGKV1-33		IGKJ4	IGKC	62974	601
SD6	P4	Plasma	94.9	100	IGH	IGHV1-46		IGHJ4	IGHG3	12624	135
SD6	P4	Plasma	97.6	90	IGK	IGKV3-20		IGKJ4	IGKC	63132	483
SD7	UN	Transitional	97.3	100	IGH	IGHV4-34		IGHJ6	IGHG1	13788	125
SD7	UN	Transitional	98.9	100	IGK	IGKV1-6		IGKJ4	IGKC	53006	344
SD8	P4	Plasma	96.9	94.7	IGK	IGKV3-20		IGKJ1	IGKC	46414	8604
SD8	P4	Plasma	98.3	100	IGH	IGHV5-51		IGHJ4	IGHG1	31074	6471

SD9	UN	Plasma	95.1	85.7	IGK	IGKV3D-20		IGKJ3	IGKC	55098	12839
SD9	UN	Plasma	89.7	100	IGH	IGHV1-69D		IGHJ1	IGHG1	22636	6025
SD10	UN	Plasma	97	100	IGH	IGHV4-30-4	IGHD3-22	IGHJ5	IGHG1	21066	4656
SD10	UN	Plasma	99.7	100	IGK	IGKV3-20		IGKJ1	IGKC	56698	11014
SD11	UN	Plasma	98.7	95.5	IGK	IGKV2-28		IGKJ1	IGKC	49796	774
SD11	UN	Plasma	96.6	100	IGH	IGHV3-21		IGHJ4	IGHG1	25588	506
SD12	UN	Plasma	98	100	IGH	IGHV3-23		IGHJ4	IGHG1	31322	331
SD12	UN	Plasma	98.6	94.7	IGK	IGKV1-33		IGKJ2	IGKC	24168	213
SD13	P10	Transitional	99.7	96.4	IGL	IGLV3-21		IGLJ1	IGLC1	10980	106
SD13	P10	Transitional	98.3	100	IGH	IGHV4-34		IGHJ4	IGHG3	12504	106
SD14	P10	Memory	95.8	95	IGK	IGKV3-15		IGKJ4	IGKC	34670	202
SD14	P10	Memory	92.3	100	IGH	IGHV4-30-4	IGHD4-17	IGHJ5	IGHG1	5740	45
SD15	P3	Transitional	95.2	92	IGL	IGLV1-44		IGLJ1	IGLC1	37842	317
SD15	P3	Transitional	90	83.3	IGH	IGHV5-51		IGHJ4	IGHG1	12332	128
SD16	P3 and P7	Transitional	95.6	75	IGH	IGHV3-48		IGHJ1	IGHG1	5502	68
SD16	P3 and P7	Transitional	94.1	75	IGL	IGLV3-21		IGLJ3	IGLC1	NA	NA
SD17	P7	Transitional	92.9	100	IGH	IGHV3-23		IGHJ3	IGHG1	3878	39
SD17	P7	Transitional	98	100	IGL	IGKV4-1		IGKJ2	IGKC	NA	NA
SD18	P4	Transitional	96.9	100	IGH	IGHV5-51		IGHJ4	IGHG1	12464	123

SD18	P4	Transitional	98.3	95.8	IGL	IGLV2-14		IGLJ3	IGLC1	NA	NA
SD19	P7	Memory	98.3	100	IGH	IGHV3-9		IGHJ6	IGHG1	13484	97
SD19	P7	Memory	98.6	100	IGL	IGLV2-14		IGLJ3	IGLC1	NA	NA
SD20	P7	Memory and transitional	97.3	100	IGH	IGHV4-4	IGHD3-10	IGHJ6	IGHG1	3892	41
SD20	P7	Memory and transitional	97.6	100	IGL	IGLV3-25		IGLJ2	IGLC1	NA	NA
SD21	P7	Switched	96	100	IGH	IGHV3-9		IGHJ4	IGHG1	1014	8
SD21	P7	Switched	97.3	100	IGL	IGLV1-51		IGLJ1	IGLC1	NA	NA
SD22	P3	Plasma	93.9	75	IGH	IGHV3-30		IGHJ4	IGHG1	394	5
SD22	P3	Plasma	95.1	100	IGL	IGKV1-33		IGKJ4	IGKC	NA	NA
SD23	P7	Transitional	93.9	100	IGH	IGHV3-66		IGHJ5	IGHG1	3350	27
SD23	P7	Transitional	99	95.5	IGL	IGKV1-27		IGKJ1	IGKC	NA	NA

[a]NA: Not available.

Table S2 The neutralization efficacy test of the expressed antibodies using qRT-PCR.

	Batch 1[a]		Batch 2		Batch 3	
Number	100 $TCID_{50}$	10 $TCID_{50}$	100 $TCID_{50}$	10 $TCID_{50}$	100 $TCID_{50}$	10 $TCID_{50}$
SD1	18.14	33.99	/	/	/	/
SD2	20.06	/	/	/	/	/
SD3	21.61	/	/	/	/	/
SD4	30.22	ND	24.51	29.62	28.75	37.21
SD5	27.35	37.14	/	/	36.31	ND
SD6	19.11	ND	/	/	/	/
SD7	37.29	21.73	/	/	17.62	ND
SD8	ND	34.02	26.11	26.2	16.08	16.92
SD9	16.58	20.86	/	/	/	/
SD10	25.2	29.1	/	/	/	/
SD11	22.39	20.42	/	/	/	/
SD12	21.71	34.33	/	/	28.45	ND
SD13	17.99	23	/	/	/	/
SD14	32.06	29.7	/	/	/	/
SD15	ND	36.61	19.4	23.21	18.16	27.49
SD17	16.79	26.58	/	/	/	/
SD18	/	/	/	/	/	/
SD19	15.92	22.56	/	/	/	/
SD20	25.68	ND	/	/	/	/
SD21	17.03	22.07	/	/	/	/
SD22	ND	36.79	24.25	25.07	21.62	28.06
SD23	18.52	ND	/	/	/	/
SF5	16.89	18.51	/	/	/	
Virus	17.17	/	13.24	16.15	13.48	16.61
Vero cells	ND	/	ND	/	ND	/

[a] ND: Undetectable, "/": not available.

Table S3 Crystallographic data collection and refinement statistics.

	SD4-Gn	SD22-Gn	SD12-Gn
**Data collection**			
Space group	*P2*	*C2*	*P21*
Cell dimensions			
a, *b*, *c* (Å)	96.856, 46.360, 97.322	64.471, 70.346, 177.469	96.064, 82.342, 125.293
α, β, γ (°)	90.0000, 113.302, 90.0000	90.0000, 90.177, 90.0000	90.0000, 104.310, 90.0000
Resolution (Å)	50.00-3.30 (3.42-3.30)[a]	50.00-2.80 (2.90-2.80)a	50.00-2.40 (2.49-2.40)[a]
Unique reflections	11858	19950	74450
R_{merge} [b]	0.243 (0.591)[a]	0.186 (1.299)a	0.161 (1.013)[a]
R_{pim}[c]	0.120 (0.354)[a]	0.091 (0.613)a	0.069 (0.486)[a]
$I/\sigma I$	5.675 (2.048)[a]	12.322 (2.072)a	11.105 (1.480)[a]
$CC_{1/2}$	0.971 (0.678)[a]	0.996 (0.749)a	0.979 (0.614)[a]
Completeness (%)	97.4 (98.3)[a]	99.7 (99.9)a	99.9 (99.9)[a]
Redundancy	4.5 (4.4)[a]	5.4 (5.6)a	6.0 (5.1)[a]
**Refinement**			
Resolution (Å)	32.21-3.30	41.94-2.78	45.92-2.40
No. reflections	11716	16920	66789
R_{work} / R_{free}[c]	0.2500/0.2887	0.3040/0.3122	0.2254/0.2552
No. atoms			
Protein	5714	4195	11352
Ligand/ion	67	67	120
Water	-	-	337
B-factors			
Protein	77.86	43.73	47.06
Ligand/ion	90.53	54.90	57.94
Water	-	-	41.06
R.m.s. deviations			

Bond lengths (Å)	0.004	0.003	0.004
Bond angles (°)	0.789	0.710	0.712
Ramachandran plot			
Favored (%)	91.04	95.04	95.77
Allowed (%)	8.41	4.41	3.89
Outliers (%)	0.54	0.55	0.34

[a]Values in parentheses are given for the highest resolution shell.

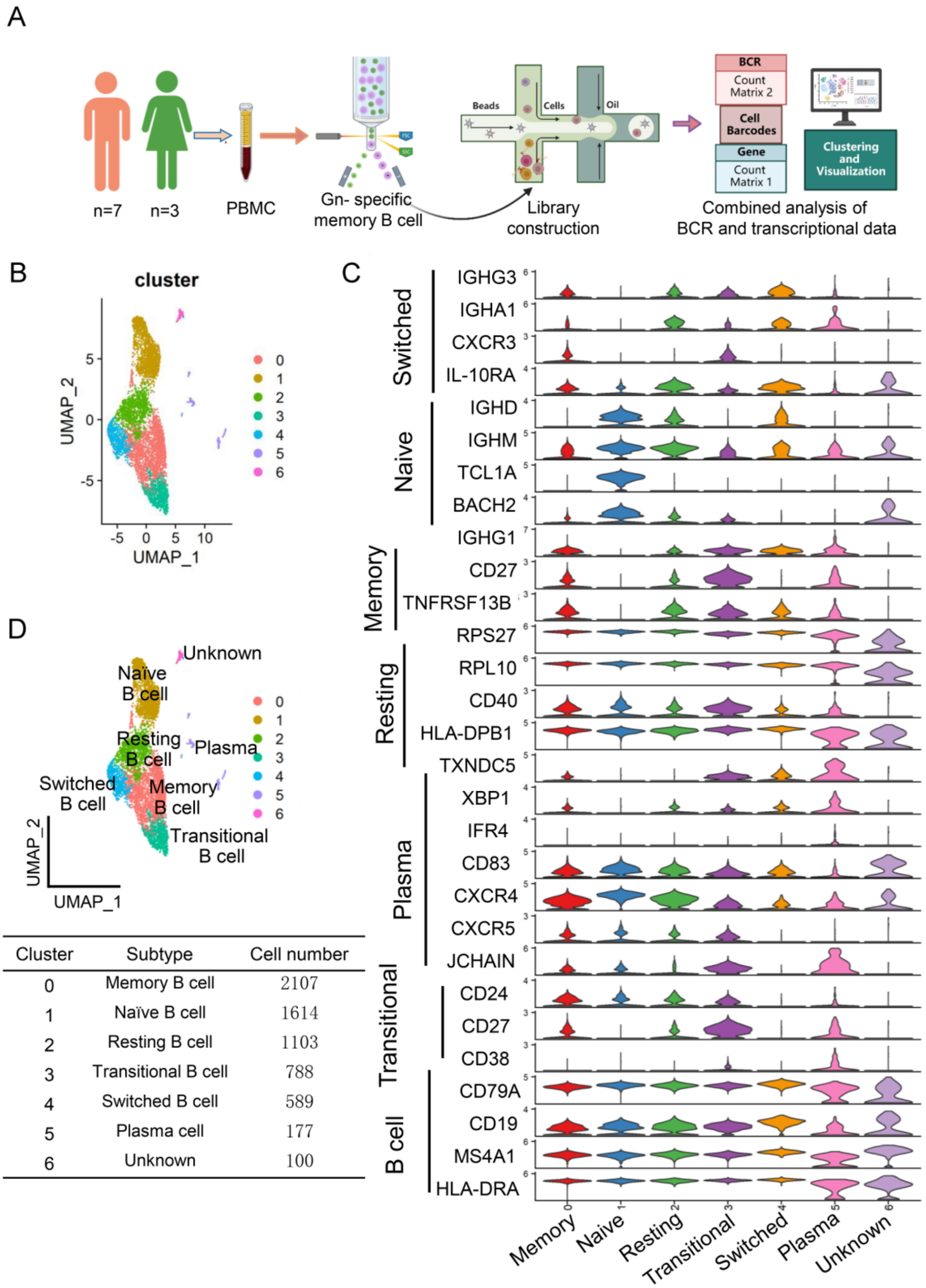

Cluster	Subtype	Cell number
0	Memory B cell	2107
1	Naïve B cell	1614
2	Resting B cell	1103
3	Transitional B cell	788
4	Switched B cell	589
5	Plasma cell	177
6	Unknown	100

FIG S1 The study design and single-cell transcriptional profiling of SFTSV-Gn specific B cells from recovered patients.

(A) A schematic diagram showing the study design to identify potential neutralizing antibodies. The SFTSV-Gn specific B cells were sorted for single-cell RNA sequencing and BCR repertoires. The BCR profiling and transcriptional expression analyses were combined based on the barcode information.

(B) The transcriptional profiling of SFTSV-Gn specific B cells was projected with uniform manifold approximation and projection (UMAP) plots with colors and seven cell clusters identified.

(C) Violin plots showing the expression distribution of the selected canonical B cell markers in the seven clusters.

(D) Identified B cell subpopulations. The UMAP projection of 6,478 single B cells was obtained from recovered patients, showing the formation of six clusters with respective labels. Each dot represents a single cell, colored according to cell type.

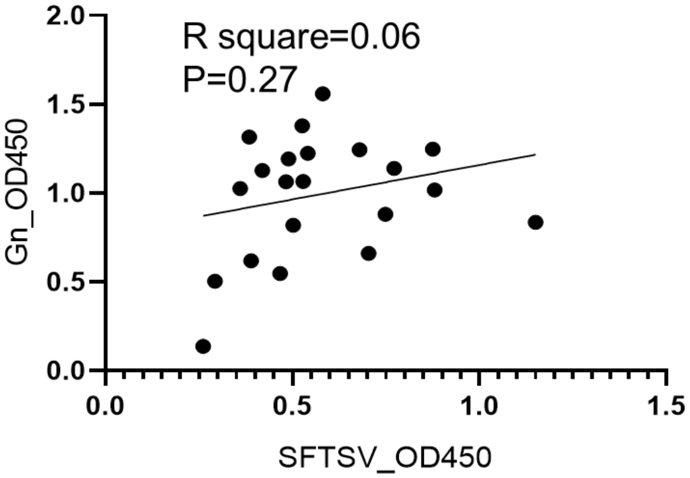

FIG S2 The correlation of expressed mAbs against SFTSV-Gn protein and SFTSV.

Two-tailed *P* values were generated by calculating Spearman's rank correlation coefficients.

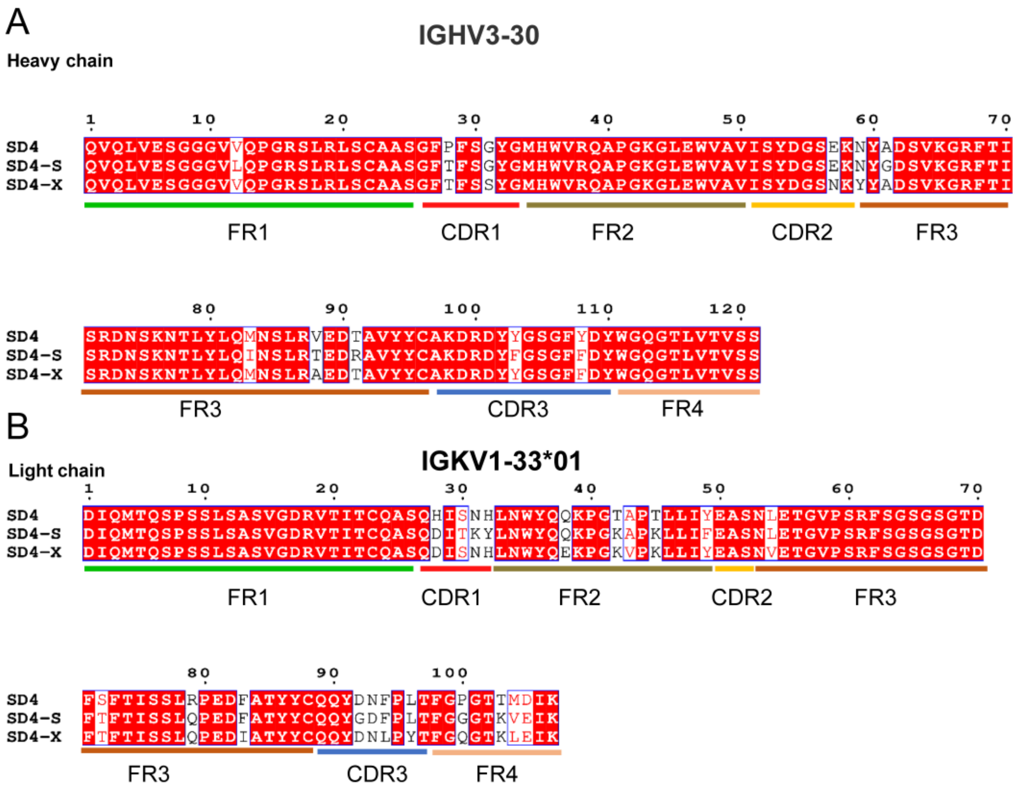

FIG S3 The sequence alignment of SD4 derived antibodies including both heavy and light chains.

Multiple sequence alignment was performed using Clustal W method embedded in Mega and showed by ESPript 3.0.

(A) The heavy chain of SD4 derived antibodies was of the IGHV3-30 genotype.

(B) The light chain of SD4 derived antibodies was of the IGKV1-33*01 genotype. The SD4 and SD4-S antibodies were isolated from P7, and the SD4-X antibody was obtained from P10.

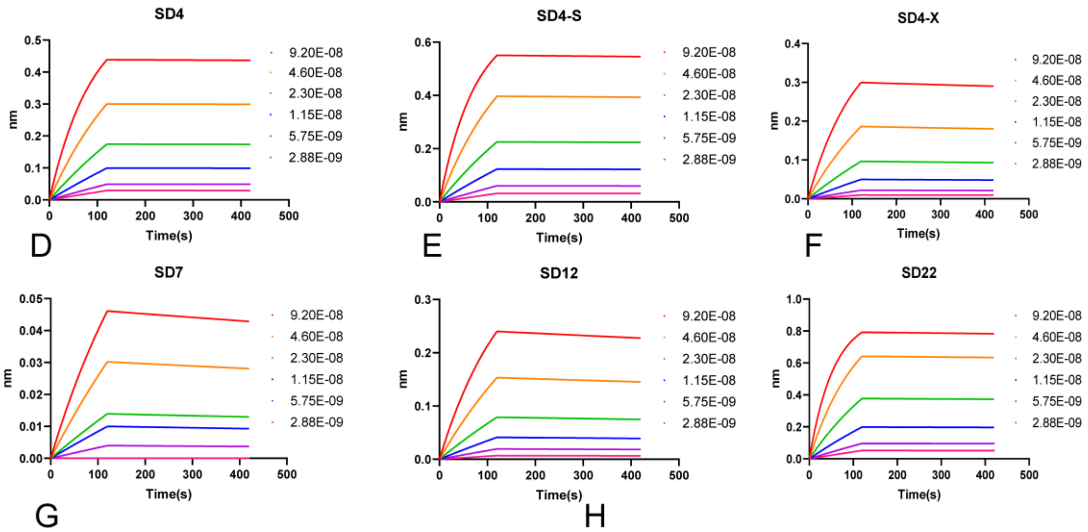

G

mAb	K_d (M)	K_{on} (1/Ms)	K_{off} (1/s)
SD4	9.630E-11	1.222E05	1.177E-05
SD4-S	1.956E-10	1.481E05	2.897E-05
SD4-X	1.107E-09	9.750E04	1.079E-04
SD7	5.296E-09	4.614E04	2.444E-04
SD12	2.368E-09	7.581E04	1.795E-04
SD22	2.051E-10	1.956E05	4.011E-05
SF5	1.213E-09	1.015E05	1.231E-04

H

mAb#	SD4	SD4-S	SD4-X	SD7	SD12	SD22	SF5
SD4	0.0113	0.0071	0.0086	0.0575	0.0024	0.0160	0.0119
SD4-S	0.0270	0.0132	0.0155	0.0736	0.0200	0.0310	0.0107
SD4-X	0.0268	0.0168	0.0154	0.0775	0.0181	0.0256	0.0101
SD7	0.0429	0.0258	0.0291	0.0180	0.0366	0.0446	0.0170
SD12	0.0178	0.0078	0.0084	0.0743	0.0100	0.0216	0.0016
SD22	0.0057	0.0051	0.0086	0.0619	0.0056	0.0127	0.0074
SF5	0.0539	0.0291	0.0304	0.0828	0.0372	0.0502	0.0236

I

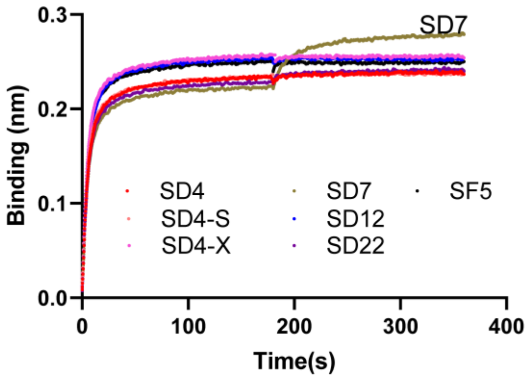

FIG S4 Binding affinity analysis of the interaction between the mAbs and the SFTSV-Gn glycoprotein.

(A-F) Binding affinities of neutralizing mAbs (SD4, SD4-S, SD4-X, SD7, SD12, and SD22) to the SFTSV Gn glycoproteins were measured by BLI.

(G) Binding kinetics (K_D, K_{on} and K_{off} constants) of antibodies with SFTSV-Gn glycoproteins are shown. Binding kinetics were obtained using the 1:1 binding kinetics fitting model employing the ForteBio data analysis software.

(H and I) Human-derived mAbs from different individuals targeted conserved epitopes on SFTSV-Gn surface antigens. The mAbs were determined to compete for the same antigenic site if the maximum binding of the second antibody was reduced to 33% compared to uncompeted binding. Competition study among neutralizing mAbs. AR2G sensors were loaded with Gn glycoprotein and subsequently saturated with SD4 or buffer. For the second antibody, the other mAbs were loaded and monitored for the additional binding capacity by Octet.

Sequence	Charge	Start	End	SD4	SD7	SD12	SF5
YVGQRGGRSQVSYYPAENS	3	70	88	-9 (2)	-5 (2)	-9 (2)	-11 (2)
YVGQRGGRSQVSYYPAENSY	3	70	89	-9 (2)	-5 (2)	-9 (1)	-13 (1)
YVGQRGGRSQVSYYPAENSYSR WSGL	4	70	95	-6 (2)	-3 (2)	-4 (1)	-10 (1)
YVGQRGGRSQVSYYPAENSYSR WSGLLSP	4	70	100	-5 (2)	-3 (2)	-4 (0)	-9 (0)
CSSDSGTSSGLLPSD	2	156	170	-15 (1)	-7 (2)	-9 (2)	-4 (3)
LMTPIPEETF	1	182	191	-1 (4)	-7 (3)	1 (2)	-1 (2)
LMTPIPEETF	2	182	191	-3 (4)	-2 (5)	-1 (1)	-2 (5)

FIG S5 The epitopes of the neutralizing mAbs were determined by mass spectrometry.

Epitopes predicted by cross-linker assisted mass spectrometry are shown in brown background, and the smaller values highlighted by heavy-brown indicate stronger binding interaction.

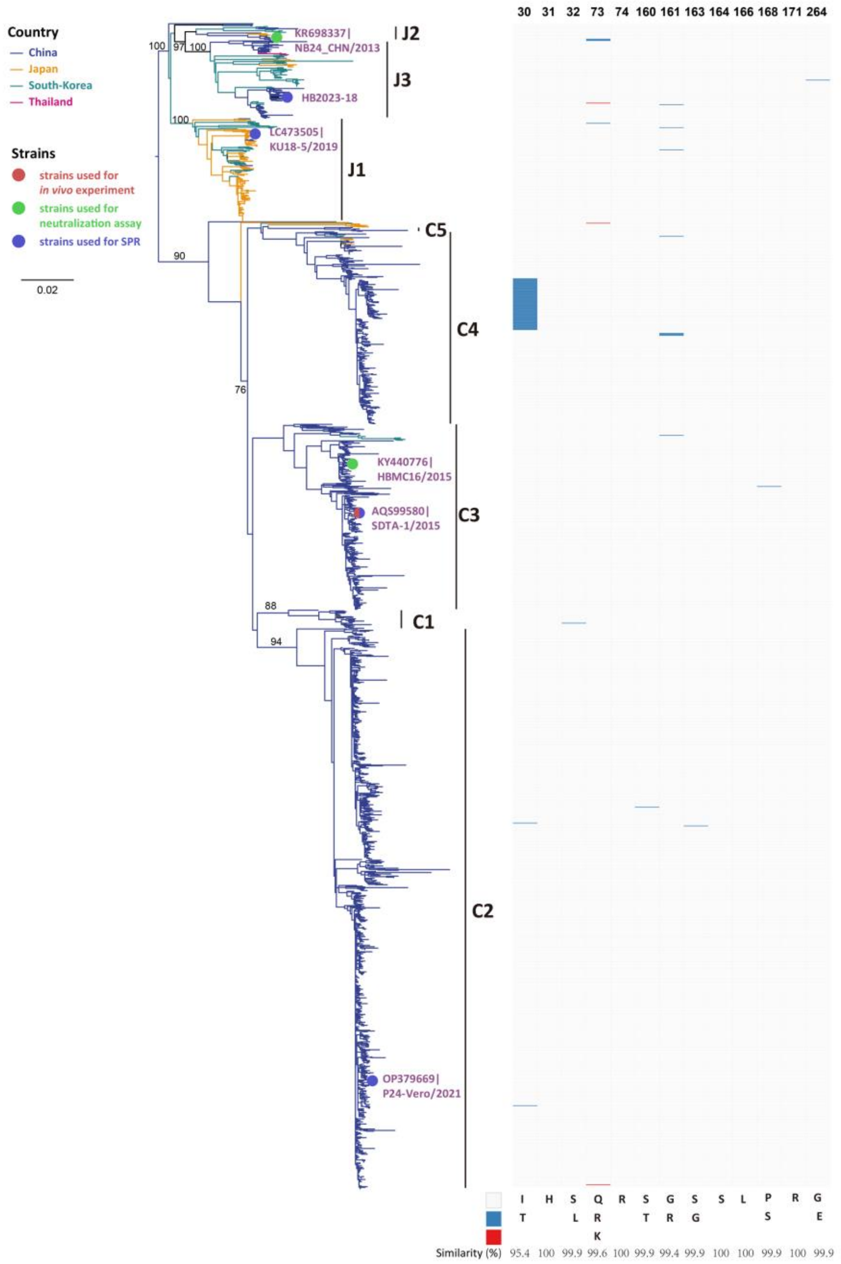

FIG S6 Phylogenetic analyses of the M gene sequences of SFTSV.

Maximum likelihood phylogenies were constructed based on 1,618 SFTSV M gene sequences available from GenBank. The SFTSV strains isolated from different countries were highlighted with distinct colors. The SFTSV strains used in the neutralization test, BLI, and animal experiments were marked with green, blue, and red cycles, respectively. The 13 amino acids commonly recognized by SD4 and SD12

antibodies were aligned from 1,618 SFTSV M gene sequences for conservation analyses. The similarity rates of these 13 binding sites were listed below.

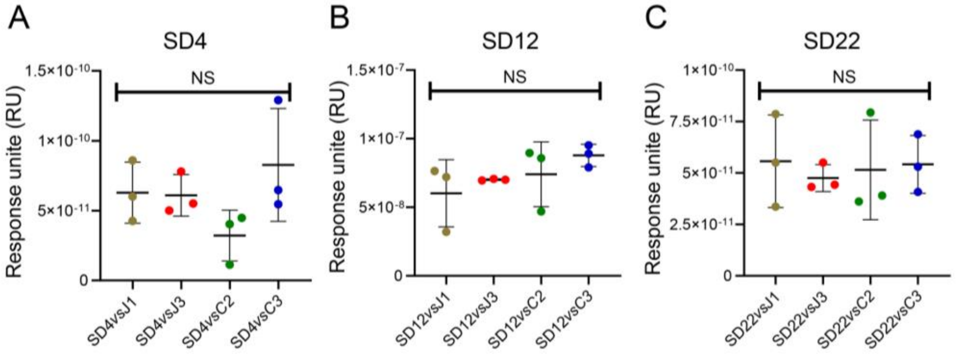

FIG S7 The binding ability of different neutralizing antibodies to SFTSV-Gn of different genotypes.

(A-C) The binding capability of SD4, SD12, and SD22, were compared with SFTSV-Gn (GenBank:LC473505, J1 genotype; HB2023-18, J3 genotype; GenBank: OP379669, C2 genotype; GenBank: AQS99580, C3 genotype), respectively. Brown-Forsythe ANOVA tests with Holm-Sidak's multiple comparisons test was used for the analyses. The mean with SD is shown for each group, and no significant difference was found.

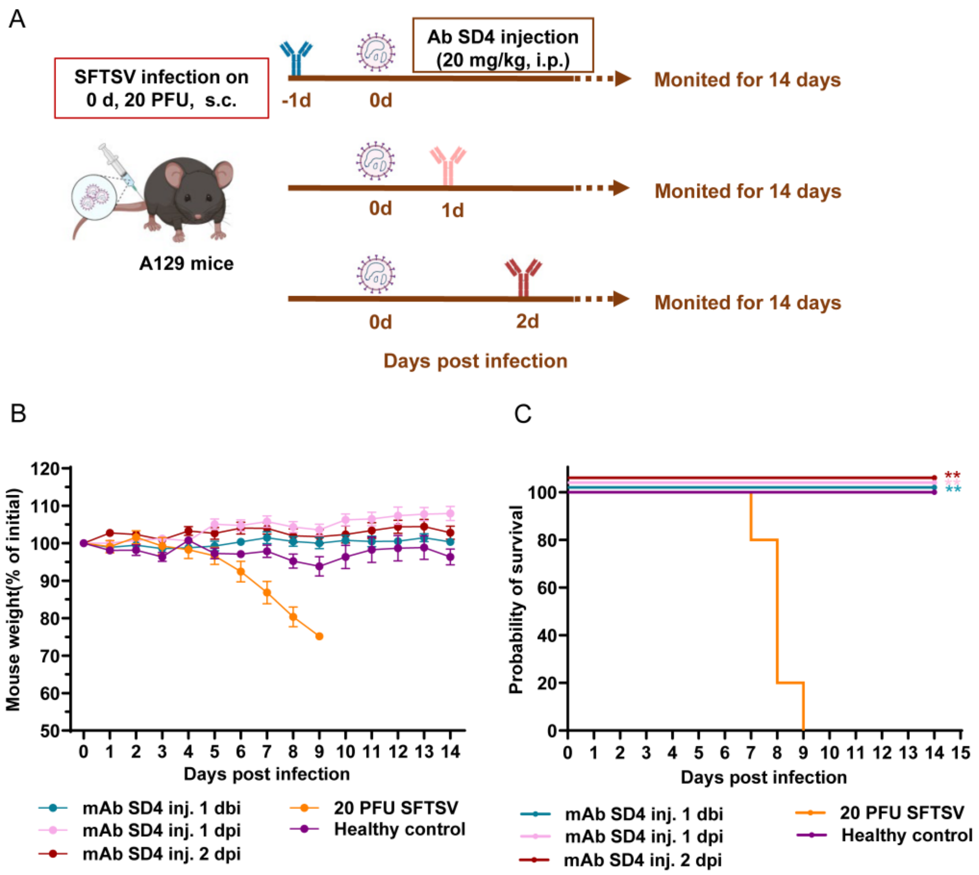

FIG S8 Protective efficacy of SD4 in type I interferon receptor-deficient (IFNα/βR$^{-/-}$) A129 mice.

(A) The experimental design. The administration of antibody SD4 was employed for both prophylaxis and early treatment. For pre-exposure treatment, mice were injected with SD4 (20 mg/kg), followed by the challenge with SFTSV 1 day later. At 24 and 48 h post-infection, infected mice were intraperitoneally administered with 20 mg/kg of SD4.

(B and C) Percentages of body weight change relative to the day of virus inoculation and survival rate were monitored daily until 14 dpi.

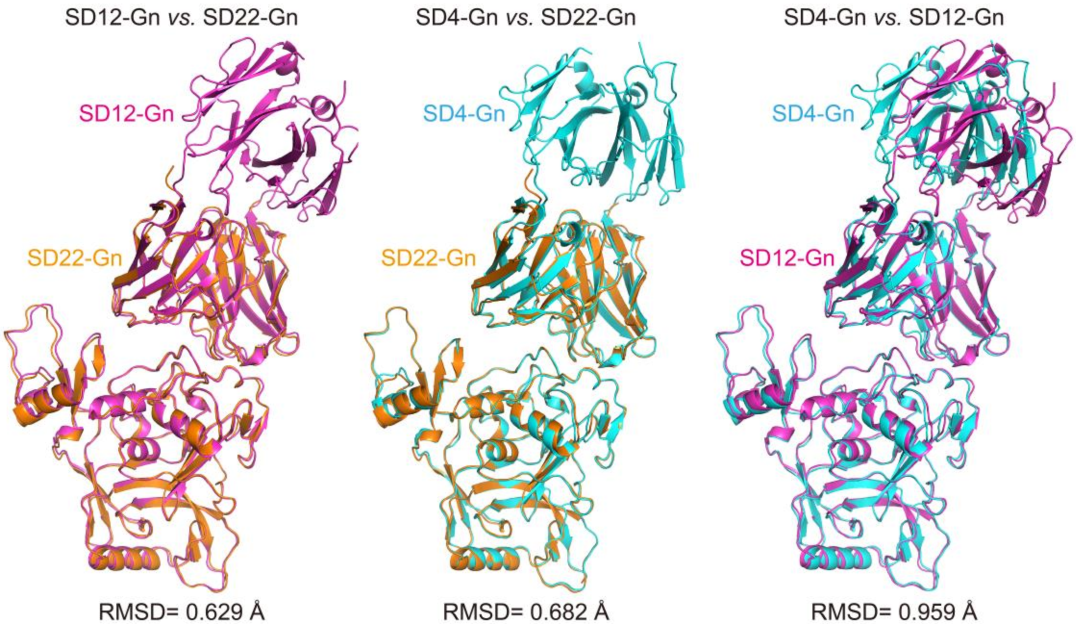

FIG S9 Structural alignment of SD4-Gn, SD22-Gn, and SD12-Gn complexes.

Structural superimpositions of the antibody-Gn complexes are shown: SD12-Gn versus SD22-Gn (left), SD4-Gn versus SD22-Gn (middle), and SD4-Gn versus SD12-Gn (right). SD4-Gn, SD22-Gn, and SD12-Gn are represented in cyan, orange, and magenta, respectively. The RMSD values are indicated.

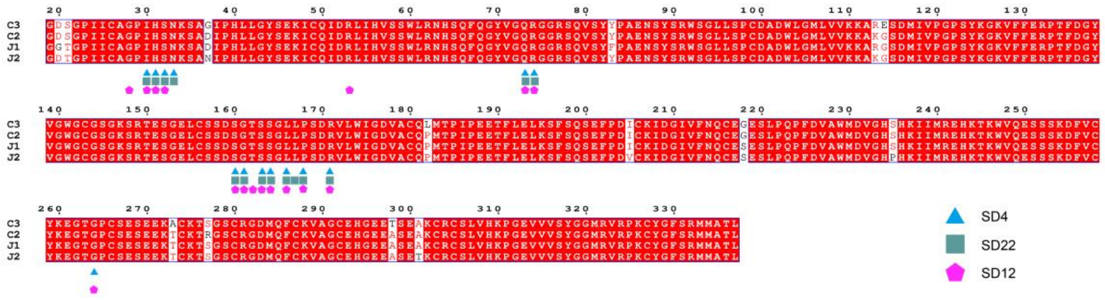

FIG S10 Protein sequence alignment of the SFTSV-Gn head from of four genotypes.

The triangles, squares, and pentagram indicate amino acids in Gn that bind to SD4, SD22, and SD12, respectively.

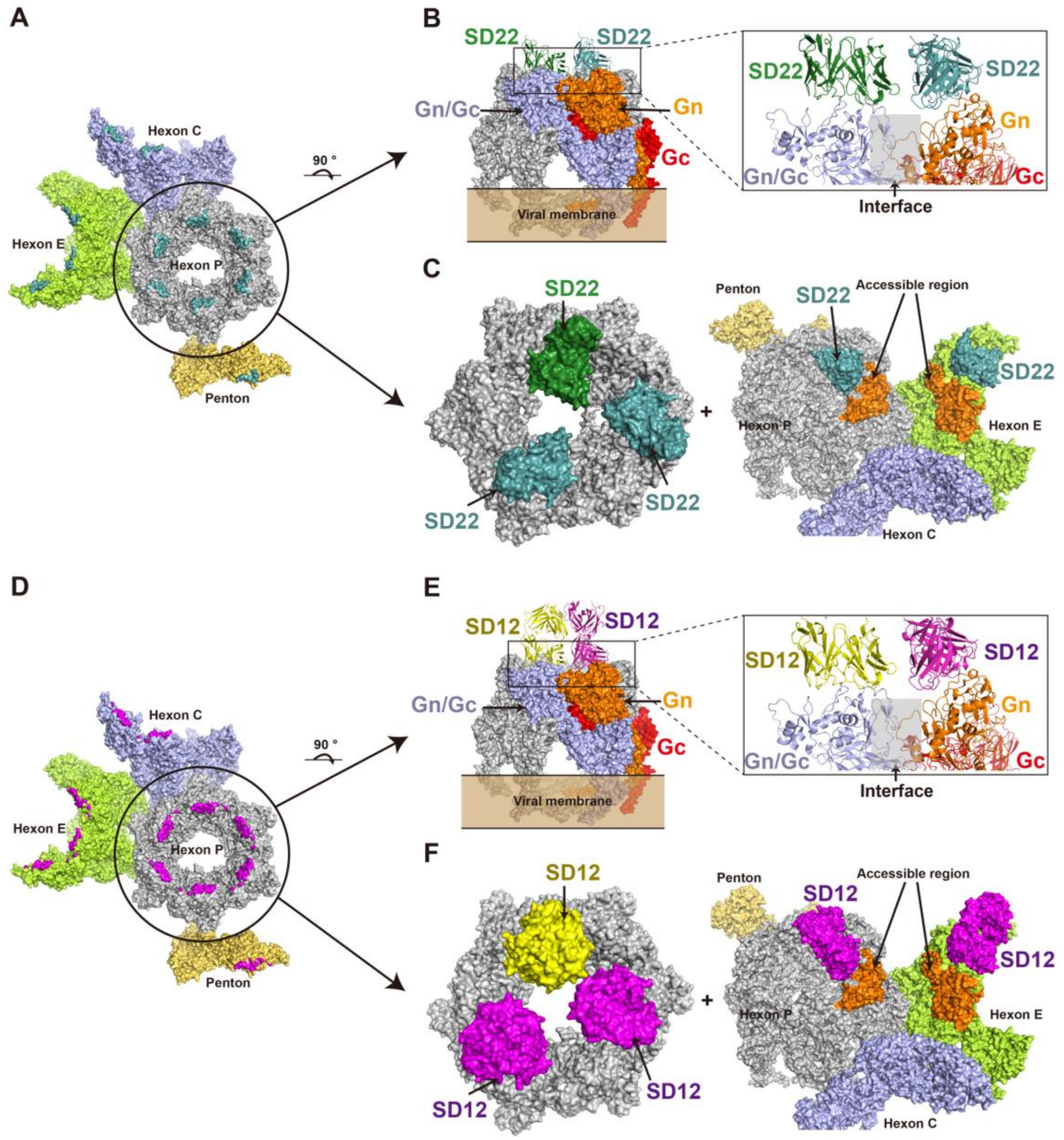

FIG S11 SD22 and SD12 epitopes on the Gn protein in the SFTSV virion.

(A and D) Epitope mapping on the SFTSV virion (PDB: 8I4T). The SD22 (A) and SD12 (D) epitopes on the virion are highlighted in light teal and magenta, respectively. (B and E) Superimposed structure of two SD22-Gn (B) and SD12-Gn (E) complexes with a hexon unit from the virion. The Gn, Gc, and Gn/Gc regions are colored orange, red, and light blue, respectively. The SD22 (light teal and forest) and SD12 (magenta and yellow) antibodies are shown as cartoon.

(C and F) Top view of the superimposed structure of three SD22-Gn (C) and SD12-Gn (F) complexes bound to a hexon unit of the virion. Side view of the superimposed structure showing two SD22-Gn and SD12-Gn complexes bound to a hexon unit and an adjacent hexon unit of the virion. The SD22 (light teal and forest) and SD12 (magenta and yellow) antibodies are represented as surface, with the accessible region highlighted in orange.

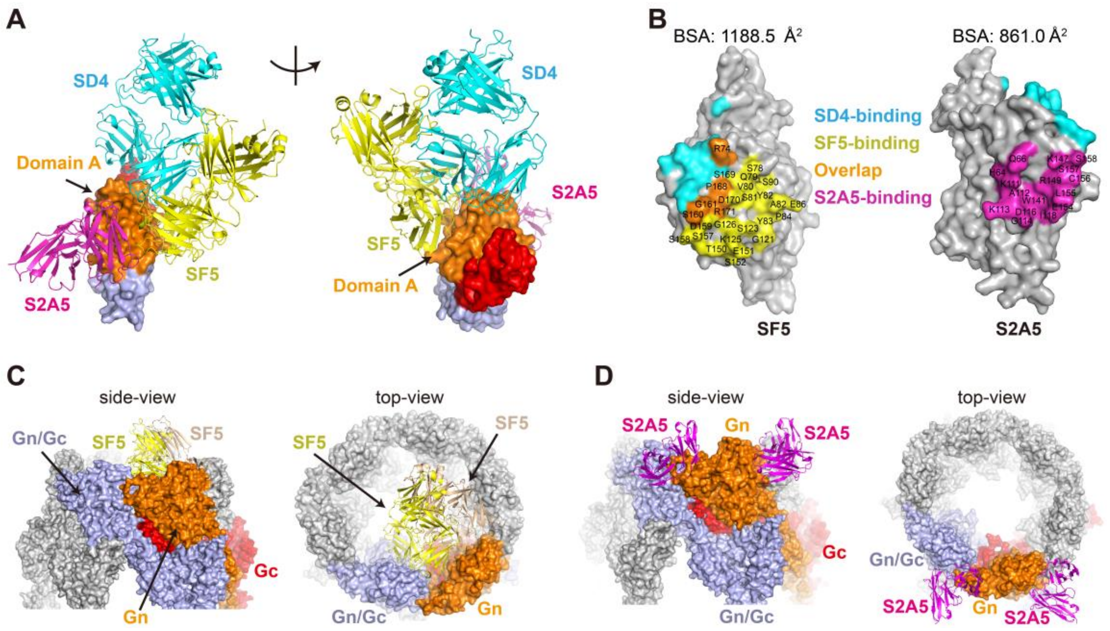

FIG S12 Binding of SF5, and S2A5 to the Gn protein in the SFTSV virion.

(A) Structural alignment of the Gn in complex with SD4, SF5, or S2A5. The SD4, SF5, and S2A5 antibodies are highlighted in cyan, yellow, and magenta, respectively, while domain A of Gn is shown in orange.

(B) Surface representation of the contact residues on Gn interacting with SF5 (left) and S2A5 (right). The binding residues on Gn interacting with SD4, SF5, and S2A5 are highlighted in cyan, yellow, and magenta, respectively. The overlap binding of SD4 with SF5 are colored in orange. The buried surface areas of the Gn-antibody interfaces are also shown.

(C) Superimposed structure of three SF5-Gn complexes with a hexon unit from the virion. The Gn, Gc, and Gn/Gc regions are colored orange, red, and light blue, respectively. The SF5 antibodies are shown in wheat and yellow.

(D) Superimposed structure of two S2A5-Gn complexes with a hexon unit from the virion. The Gn, Gc, and Gn/Gc regions are colored orange, red, and light blue, respectively. The S2A5 antibodies are shown in magenta.
